# Deep structured learning with vision intelligence for oral carcinoma lesion segmentation and classification using medical imaging

**DOI:** 10.1038/s41598-025-89971-5

**Published:** 2025-02-24

**Authors:** Ahmad A. Alzahrani, Jamal Alsamri, Mashael Maashi, Noha Negm, Somia A. Asklany, Abdulwhab Alkharashi, Hassan Alkhiri, Marwa Obayya

**Affiliations:** 1 Department of Computer Science and Artificial Intelligence, College of Computing, Umm-AlQura University, Mecca, Saudi Arabia; 2 Department of Biomedical Engineering, College of Engineering, Princess Nourah bint Abdulrahman University, Riyadh, Saudi Arabia; 3 Department of Software Engineering, College of Computer and Information Sciences, King Saud University, PO Box 103786, 11543 Riyadh, Saudi Arabia; 4 Department of Computer Science, Applied College at Mahayil, King Khalid University, Abha, Saudi Arabia; 5 Department of Computer Science and Information Technology, Faculty of Sciences and Arts, Turaif, Northern Border University, 91431 Arar, Saudi Arabia; 6 Department of Computer Science, College of Computing and Informatics, Saudi Electronic University, Riyadh, Saudi Arabia; 7 Department of Computer Science, Faculty of Computing and Information Technology, Al-Baha University, Al-Baha, Saudi Arabia

**Keywords:** Deep learning, Oral carcinoma, Weiner filtering, Sine cosine algorithm, Medical imaging, Computer science, Information technology

## Abstract

Oral carcinoma (OC) is a toxic illness among the most general malignant cancers globally, and it has developed a gradually significant public health concern in emerging and low-to-middle-income states. Late diagnosis, high incidence, and inadequate treatment strategies remain substantial challenges. Analysis at an initial phase is significant for good treatment, prediction, and existence. Despite the current growth in the perception of molecular devices, late analysis and methods near precision medicine for OC patients remain a challenge. A machine learning (ML) model was employed to improve early detection in medicine, aiming to reduce cancer-specific mortality and disease progression. Recent advancements in this approach have significantly enhanced the extraction and diagnosis of critical information from medical images. This paper presents a Deep Structured Learning with Vision Intelligence for Oral Carcinoma Lesion Segmentation and Classification (DSLVI-OCLSC) model for medical imaging. Using medical imaging, the DSLVI-OCLSC model aims to enhance OC’s classification and recognition outcomes. To accomplish this, the DSLVI-OCLSC model utilizes wiener filtering (WF) as a pre-processing technique to eliminate the noise. In addition, the ShuffleNetV2 method is used for the group of higher-level deep features from an input image. The convolutional bidirectional long short-term memory network with a multi-head attention mechanism (MA-CNN‐BiLSTM) approach is utilized for oral carcinoma recognition and identification. Moreover, the Unet3 + is employed to segment abnormal regions from the classified images. Finally, the sine cosine algorithm (SCA) approach is utilized to hyperparameter-tune the DL model. A wide range of simulations is implemented to ensure the enhanced performance of the DSLVI-OCLSC method under the OC images dataset. The experimental analysis of the DSLVI-OCLSC method portrayed a superior accuracy value of 98.47% over recent approaches.

## Introduction

Cancer is a leading public health difficulty and the 2nd common reason for death in advanced countries. OC is among the ten typical cancers; above 90% are squamous cell carcinomas^1^. Regardless of therapeutic and diagnostic growth in OC patients, morbidity and mortality rates have stayed higher with no development in the past 50 years, mainly owing to the last phase of diagnosis when metastatic cancer appeared^2^. Regularly, oral squamous cell carcinoma (OSCC) occurs from previous lesions of oral mucosa with an improved threat for malignant metamorphosis in cancer. Usually, OSCC can be treated mainly through surgical sections with or without adjunct radiation, which primarily influences the patient’s standard of living^3^. Successful detection of patient results, successful producibility and objectivity, correspondingly reducing intra-and inter-observer inconsistency using the Artificial Intelligence (AI) models, can directly influence personalized treatment intervention by so it might help the pathologist to lessen a load of physical examinations along with making faster results with high accuracy. The earlier examination is essential for better survival, prognosis, and treatment^4^. Late diagnosis complicates the development of accurate treatment plans, although newer growths exist in understanding the tumour’s molecular system. Computer-aided diagnosis (CAD), recognition, and medical imaging techniques are now used to identify potential variations in cancer treatment. These methods enable the early detection of cancer by analyzing X-ray images, computed tomography (CT) scans, and magnetic resonance imaging (MRI) scans^5^.

These techniques facilitate the anatomical study of the oral cavity and enable the precise removal of cancer-prone areas. During the tumour removal phase, image analysis uses various segmentation models to differentiate between the affected areas and tumour-prone regions^6^. The usage of deep learning (DL) techniques and appropriate CT image segmentation must declare the effect of the current solutions for the precise classification and detection of OC. Developments in the domains of DL and computer vision (CV) present efficient models to grow adjuvant techniques that can carry out an automatic oral cavity screening and offer a response to medical professionals throughout patient check-ups in addition to individuals for self-analysis^7^. The studies on image-based automatic OC analysis have mainly concentrated on using special techniques like hyperspectral imaging, autofluorescence imaging, and optical coherence tomography. Instead, a brief research study uses white-light photographic images, primarily focusing on detecting specific oral lesion types^8^. OSCC identification can be critical for increasing the earlier detection of OC and, hence, plays a significant role in developing OC screening tools^9^. Using the fast improvement in CAD in recent years, the DL application plays a substantial part in medicine. From a rapid examination of some medical modalities for diagnosing and detecting cancer in different parts of the body, DL has had extensive results in medical science^10^. Convolutional neural network (CNN) is the most effective method of DL in the medical imaging study area.

This paper presents a Deep Structured Learning with Vision Intelligence for Oral Carcinoma Lesion Segmentation and Classification (DSLVI-OCLSC) model for medical imaging. Using medical imaging, the DSLVI-OCLSC model aims to enhance OC’s classification and recognition outcomes. To accomplish this, the DSLVI-OCLSC model utilizes wiener filtering (WF) as a pre-processing technique to eliminate the noise. In addition, the ShuffleNetV2 method is used for the group of higher-level deep features from an input image. The convolutional bidirectional long short-term memory network with a multi-head attention mechanism (MA-CNN‐BiLSTM) approach is utilized for OC recognition and identification. Moreover, the Unet3 + is employed to segment abnormal regions from the classified images. Finally, the sine cosine algorithm (SCA) approach is utilized to hyperparameter-tune the DL model. A wide range of simulations is implemented to ensure the enhanced performance of the DSLVI-OCLSC method under the OC images dataset. The major contribution of the DSLVI-OCLSC method is listed below.

- The WF model is applied as a pre-processing technique to mitigate noise in input images, improving the data quality for analysis. This step confirms cleaner, more accurate feature extraction and model training. Improving image clarity directly assists the efficiency of downstream carcinoma recognition and segmentation tasks.
- The ShuffleNetV2 method extracts high-level deep features from the input images, improving the effectiveness and accuracy of feature extraction. This methodology mitigates computational complexity while maintaining robust performance in capturing relevant image patterns. Improving feature representation crucially supports the classification and segmentation tasks in carcinoma detection.
- The MA-CNN-BiLSTM approach is employed for carcinoma recognition and detection, incorporating convolutional layers with bidirectional LSTM to capture spatial and temporal dependencies. The multi-head attention mechanism improves focus on critical features, enhancing the accuracy of the classification. This integration allows for robust detection and identification of carcinoma in complex medical images.
- The Unet3 + technique is employed for precisely segmenting carcinoma lesions, enhancing the model’s ability to accurately localize lesions in cancerous regions. This methodology improves segmentation accuracy using deeper skip connections and multi-scale feature fusion. As a result, it confirms improved delineation of tumour boundaries, assisting in more reliable diagnosis and treatment planning.
- The novelty of the DSLVI-OCLSC model is its seamless integration of several advanced techniques, comprising noise reduction, feature extraction, carcinoma recognition, and segmentation. The model effectively handles complex image analysis tasks by combining WF, ShuffleNetV2, MA-CNN-BiLSTM, and Unet3+. Additionally, hyperparameter tuning via the SCA further optimizes performance. This multi-faceted approach improves the accuracy and efficiency of carcinoma detection and segmentation.

## Review of literature

Rönnau et al.^11^ present a CNN for automated classification and segmentation of cells in Papanicolaou tainted images. The CNN has been evaluated and trained on a novel image cells dataset from oral mucosa interpreted by experts. The efficiency of the method has been assessed against an expert group. Its strength is also illustrated on five cervical image public datasets taken by various cameras and microscopes, taking background intensities, noise levels, resolutions, and colours. Zhang et al.^12^ introduced extensively utilized pathological segments with Hand E stains, which have been regarded as the target and integrated with the developments of hyperspectral imaging technology. A new diagnosis method for recognizing OSCC lymph node metastases is presented. The technique contains decision-making and learning phases, concentrating on non-cancer and cancer nuclei, progressively implementing the segmentation of lesions from course to fine, and attaining higher precision. In the decision-making phase, the outcomes of segmentation were post-processed, and the lesions were efficiently prominent depending upon the first. Shukla et al.^13^ present a new method which integrates machine vision for detecting cancer and targets to improve the precision of diagnosis. Considering the HI’s complex nature, the author implements unsupervised methods for detecting cancer against the supervised or conventional DL methods. The nucleus in a tumorous tissue surgery image can be recognized as the ROI owing to its essential form and features. Maia et al.^14^ presented a DL to handle the automatic recognition of different pathologies by utilizing digital images. One of the most significant restrictions for employing DL to HI denotes the absence of public datasets. A joint effort was created to stop this gap, and a novel dataset of HI of OC, called P-NDB-UFES, was gathered, explained, and studied by oral pathologists, making the gold standard for identification.

Hoda et al.^15^ presented a concise overview of evolving optical imaging AI-based methods and their implications and applications for developing OC detection. Initial OC diagnosis assists in enabling initial treatment results and predicting complete patient diagnosis. The study discusses the use of CNN for OC image classification. Additionally, the morphological processes have been utilized for cancer areas’ image-gathering segmentation. Then, the DL method has been used to distinguish the cancer lesion areas into severe or mild lesion areas. Chen et al.^16^ presented 2 DL techniques, Transformers and CNN. Initially, a novel CANet classification method for OC was proposed that utilizes attention mechanisms integrated with ignored position information to find the intricate fusion of deep networks and attention mechanisms and strike the possibility of attention mechanisms entirely. The image can be segmented into a sequence of 2D image blocks. Later, that is handled by many layers of transformation blocks. Meer et al.^17^ presented a completely automatic structure based on Self Attention CNN and ResNet information optimization and fusion. In the presented method, augmentation was performed on the testing and training models, and then two advanced deep techniques were trained. A self-attention MobileNetV2 method has been trained and developed using an amplified dataset. Separately, a Self-Attention DarkNet19 method was trained on the equivalent dataset, where the hyperparameter is modified by utilizing the WOA. Features have been extracted from both methods’ deep layers and united using a CCA method. Raval and Undavia^18^ present a DL-based technique for identifying oral and skin cancer utilizing medical images. The author discusses CNN methods like Inception, AlexNet, ResNet, VGGNet, Graph Neural Network (GNN), and DenseNet. Image processing methods like image filtering and image resizing have been utilized for OC and skin cancer images to enhance the excellence and reduce noise from images.

Dharani and Danesh^19^ introduce two DL methods, MaskMeanShiftCNN for segmenting OSCC regions using colour, texture, and shape features and SV-OnionNet for early-stage OSCC identification from histopathological images. Yang et al.^20^ evaluate DL approaches for OCT images to assist in OC screening, comparing CNN models with ML methods. Zhou et al.^21^ present SPAT_SmSL, an intelligent system for OSCC diagnosis and prognosis, utilizing Self-supervised Pretraining (SP) and Adaptive Thresholding (AT) to segment key regions and quantify prognostic factors, followed by Cox analysis for survival prediction. Haq et al.^22^ explore AI in OSCC diagnosis using Gabor + CatBoost, ResNet50 + CatBoost, and Gabor + ResNet50 + CatBoost. Features from Gabor filters and ResNet50 are extracted, optimized with PCA, and classified using CatBoost. Pinnika and Rao^23^ analyze various DL models for the segmentation and classification of OC, aiming to improve early detection and patient survival rates. Ahmad et al.^24^ propose hybrid AI methods for early OSCC diagnosis, using transfer learning (TL) with five CNNs, CNN-based feature extraction with SVM classification, and feature fusion through PCA and texture analysis (GLCM, HOG, LBP). Dutta et al.^25^ explore AI’s efficiency in early OC detection using radial basis function networks (RBFN) and stochastic gradient descent (SGDA), along with two DL techniques for recognizing oral lesions. Islam et al.^26^ aimed to use DL models, namely VGG19, DeIT, and MobileNet, for classifying oral lesions into benign and malignant categories. Albalawi et al.^27^ address the challenge of diagnosing OSCC by developing a DL approach based on the EfficientNetB3 model. Zhu et al.^28^ propose CariesNet, a DL technique for segmenting caries lesions in panoramic radiographs.

The existing studies for automated classification and segmentation of cancerous lesions have several limitations. Many models rely on expert-annotated datasets, which may not generalize well to new or unannotated data and can be influenced by discrepancies in imaging conditions, such as camera quality and noise. Techniques utilizing hyperspectral imaging or DL models, such as CNNs and transformers, can be computationally expensive and perform poorly with lower-quality images. The lack of diverse public datasets for specific cancer types limits model generalization. Furthermore, image pre-processing methods may need to fully address noise or complex lesion segmentation challenges. A key research gap is the lack of diverse and publicly available datasets for training and evaluating cancer detection models, particularly for OC, which limits the generalizability of existing methods. Many existing approaches also depend on expert annotations, which may need to scale better to real-world applications. Moreover, while DL methodologies such as CNNs and transformers exhibit promise, they mostly face discrepancies in image quality, noise, and computational efficiency, underscoring the requirement for more robust and scalable models.

## Materials and methods

**Fig. 1:**
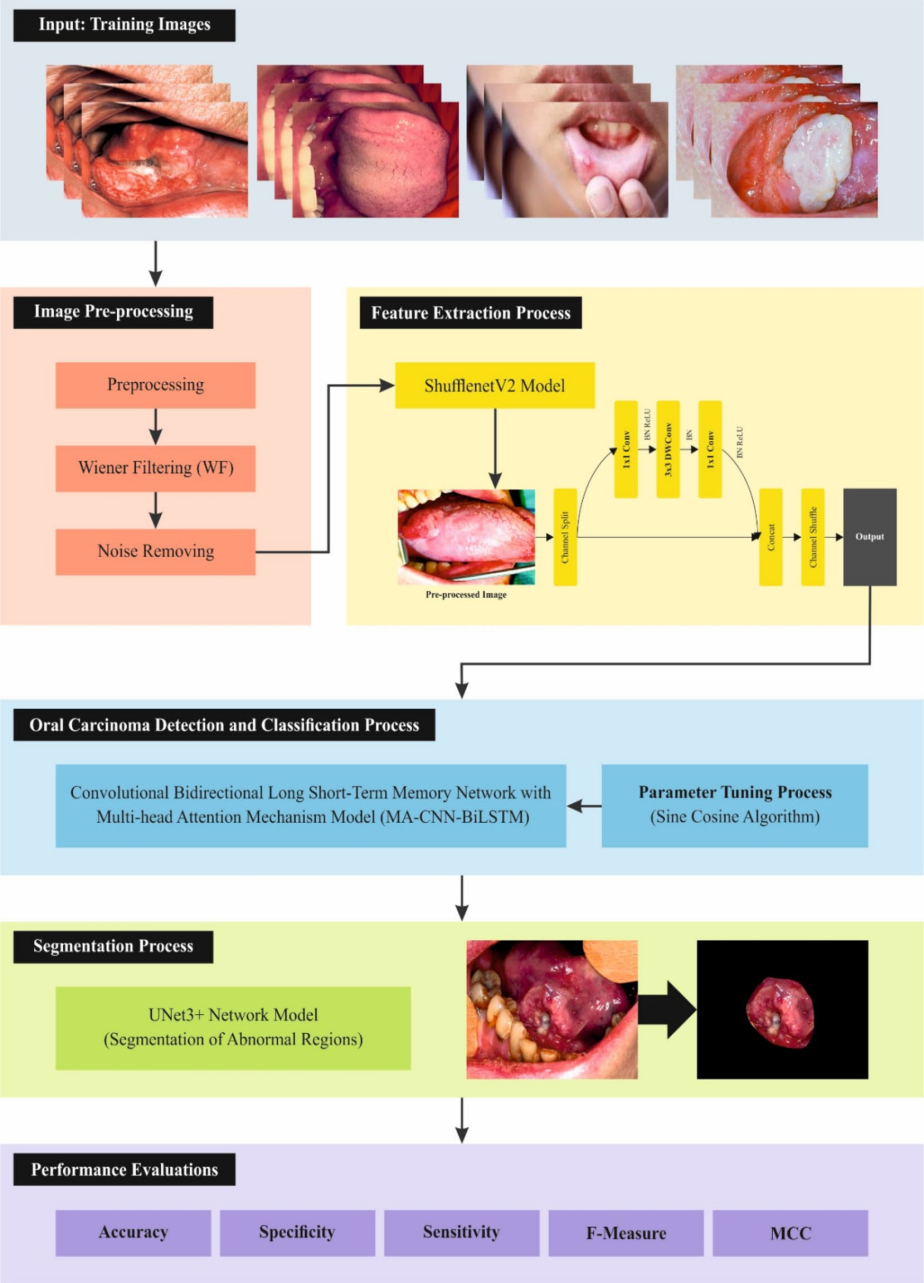
Workflow of DSLVI-OCLSC model.

In this article, a DSLVI-OCLSC model is presented for medical imaging. The main objective of the DSLVI-OCLSC model is to progress the classification and recognition outcomes of OC using medical imaging. To accomplish this, the DSLVI-OCLSC model involves five stages: pre-processing, feature extractor, classification, segmentation, and parameter tuning processes, depicted in Fig. 1.

### Pre-processing: WF technique

**Fig. 2:**
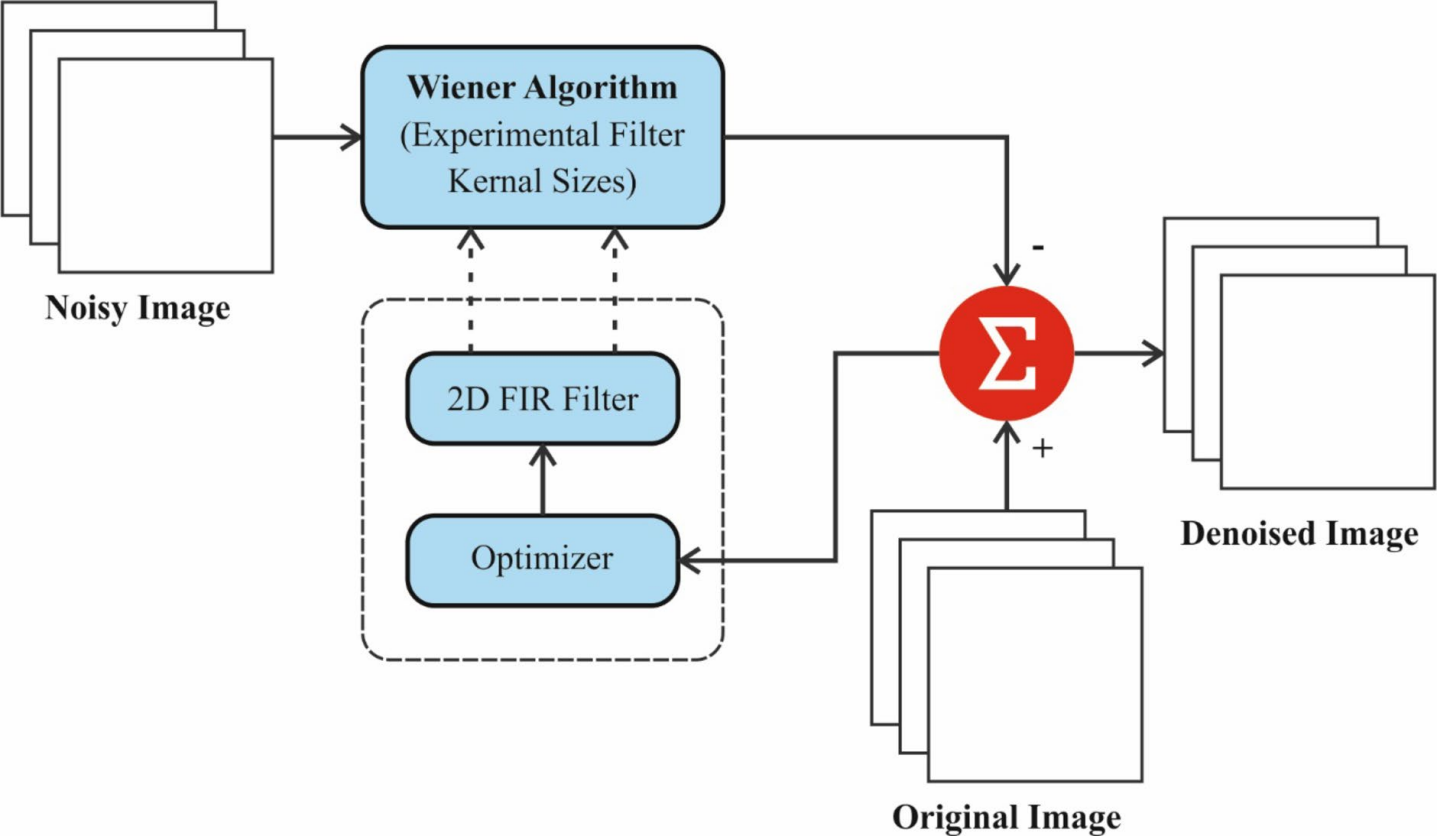
WF framework.

Primarily, the DSLVI-OCLSC model employs the WF-based pre-processing technique to eliminate the existing noise^29^. This technique is chosen because it can effectively mitigate noise in medical images while preserving crucial details. It is specifically beneficial in applications where noise reduction is critical, such as in medical imaging, where clarity and precision are significant for precise diagnosis. Unlike other denoising methods, the WF method adapts to the local noise characteristics, presenting optimal filtering performance for diverse regions of the image. Its statistical approach allows for improved handling of discrepancies in noise levels, making it highly effective for X-ray, CT, and MRI images. Additionally, it is computationally efficient, ensuring fast processing times in time-sensitive medical environments. Figure 2 demonstrates the architecture of the WF approach.

WF is an innovative image processing model that improves medical images by decreasing noise while maintaining significant features. In the framework of OC, WF is used to enhance the precision of images obtained from medical scans, such as CT or MRI, permitting improved visualization of cancer boundaries. This improvement helps in more precise analysis and treatment planning by emphasizing vital facts of the carcinoma. The filter adjusts to the local signal-to-noise percentage, certifying that the main features of OC are kept while noise is diminished.

### Feature extractor: ShuffleNetV2 approach

**Fig. 3:**
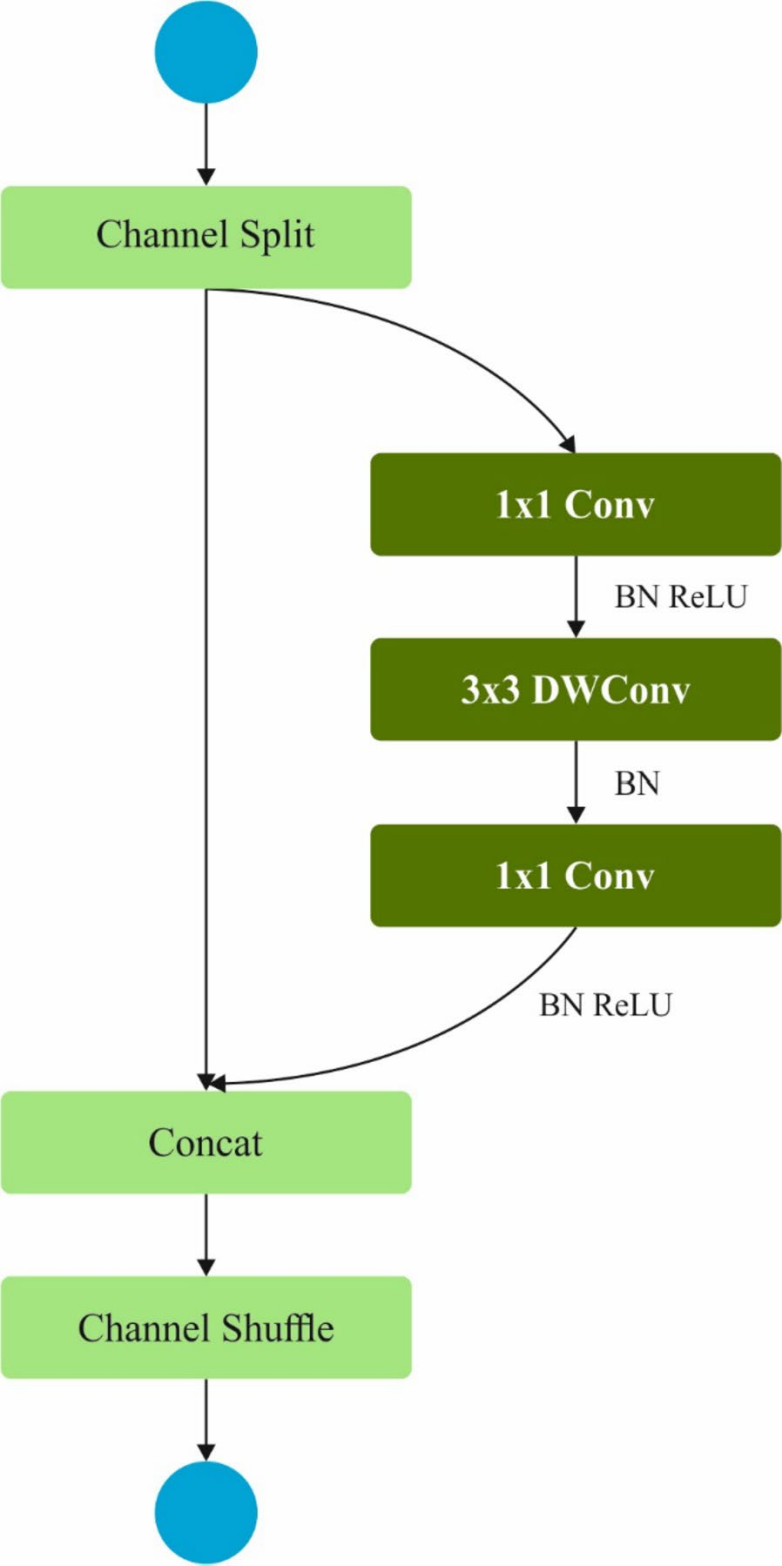
Structure of ShuffleNetV2 model.

In addition, the ShuffleNetV2 method is utilized for the group of higher-level deep features from an input image^30^. This model is chosen due to its exceptional balance between efficiency and accuracy. It is designed to perform well on mobile and embedded devices, making it ideal for real-time medical image analysis where computational resources are often limited. Unlike traditional convolutional neural networks (CNNs), the ShuffleNetV2 model employs effectual channel shuffling and group convolutions, which mitigate computational costs without losing performance. This makes it specifically appropriate for tasks that need processing massive volumes of medical images, such as tumour detection. Furthermore, its lightweight architecture ensures fast inference times, which is significant for time-sensitive applications in clinical settings. Figure 3 illustrates the structure of the ShuffleNetV2 model.

The ShuffleNet V2 model developed a lightweight method, which creates the ShuffleNet V1 structure. The succeeding four important features may impact the ShuffeNet V2 network speed. While the output and input convolutional layer channels are equivalent, the method functions at its maximal rapidity with a minimum memory access time. Unnecessary convolution (conv) operations could upsurge the memory access time, resulting in the slowest method speed. Accruing point-by-point operations could slow the method, reducing the frequencies of these extensions. It encompasses dual key modules: In the basic unit, the input features were separated consistently into dual groups below a channel split operation. The right branch consecutively negotiates the $\:1\times1$ Conv, $\:3\times 3$ depthwise Conv, and a $\:1\times1$Conv, whereas the left branch remains untreated. Consequently, the right and left branches have been concatenated, and channels have been shuffled to improve the data exchange among various groups. In the downsampling unit, the features of the image could directly enter both branches. The branch of proper endures sequential processing over a $\:1\times1$Conv, $\:3\times 3$ depthwise Conv with 2stride, and a $\:1\times1$Conv, and the branch of left initially endures $\:3\times 3$ depthwise Conv with two stride and later a $\:1\times 1$Conv. Consequently, the right and left branches have been concatenated, and the shuffling of channels improves data exchange among various groups.

### Classification: MA-CNN‐BiLSTM classifier

**Fig. 4:**
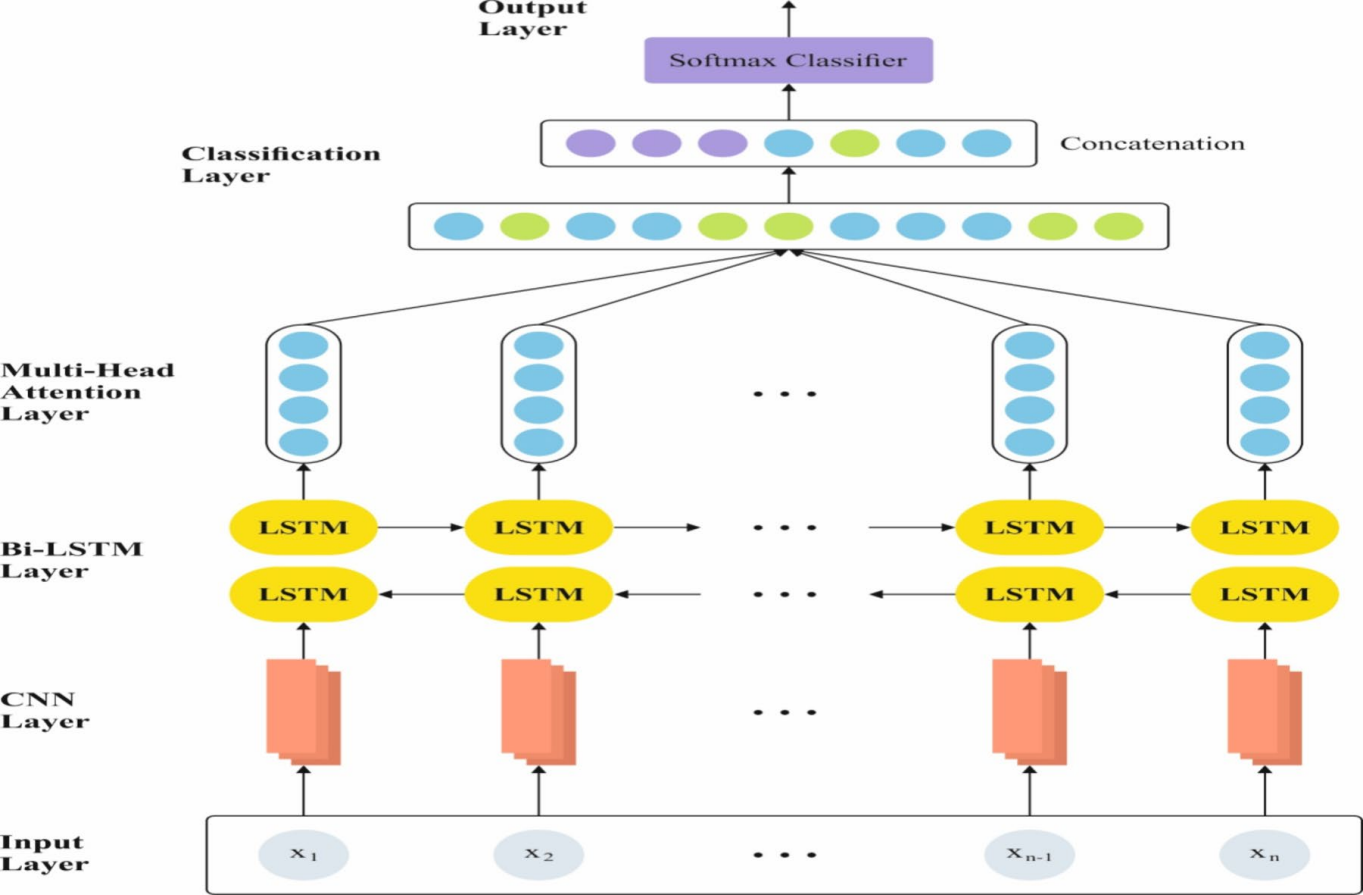
Structure of MA-CNN‐BiLSTM model.

For OC recognition and identification, the MA-CNN‐BiLSTM approach is utilized^31^. This method is chosen for its ability to efficiently capture both spatial and temporal dependencies in sequential data. The convolutional layers allow for automatic feature extraction, which is ideal for handling complex patterns in data. In contrast, the BiLSTM layers capture long-range dependencies by processing data in both forward and backward directions. Adding the multi-head attention mechanism enhances the ability of the model to concentrate on the most relevant parts of the input, improving performance in tasks with long sequences or complex relationships. This integration of CNN, BiLSTM, and attention enables the model to outperform conventional methods that might struggle with spatial feature extraction or temporal dependencies, making it a powerful choice for classification tasks. Figure 4 demonstrates the structure of the MA‐CNN‐BiLSTM model.

The current research introduced a new DL method, represented as MA-CNN‐BiLSTM that enhances the new LSTM architecture and combines MA and CNN components to achieve the main objectives of computing $\:E{T}_{0}$. It especially integrates the CNN capability to take local features and the ability of BiLSTM to study temporal dynamics and long‐term dependencies in inputs. Still, it also combines the powers of the MA method to handle complex relationships. In addition, MA‐CNN‐BiLSTM contains three main modules. The first module denotes the layer of CNN, which uses convolutional operations for feature and pattern recognition over different locations, so it effectually takes the essential local features and architectures in the input data. Consequently, the layer of the MA method, creating 2nd module, additionally improves the extraction of various relationships and features. Moreover, this layer contains numerous attention heads, where every head is proficient at concentrating on multiple sequences of input segments and studying their relationships, which provides the seizure of global contextual information and long‐range dependencies in the input sequence. Lastly, the 3rd module represents the layer of BiLSTM that is expert in taking the enduring and context dependences in input variables over backward and forward propagation. Therefore, MA‐CNN‐BiLSTM effectively removes features on various levels and perceptions, thus accurately taking contextual data. The next segment suggests a concise overview of the BiLSTM, CNN, and MA layers.

#### CNN Layer

The CNN is presented as a feedforward neural network (FFNN) model incorporating convolutional calculations. The usual CNN structure contains convolutional, input, pooling, output, and activation function layers. It focuses on convoluting the input data with various-weight convolutional kernels in the convolutional layers, enabling the removal of intrinsic features of data.

For input matrix $\:X=[{x}_{1},\:{x}_{2},\dots\:,\:{x}_{n}]$ on time node $\:t\left(t=\text{1,2},\:\dots\:,\:T\right)$ is stated as $\:{X}^{t}=\left[{x}_{1}^{t},{x}_{2}^{t},\:.,{x}_{n}^{t}\right]$, and the initial convolution layer is computed below:

1 $$\:ReLU\left(x\right)=\{X\:x>0\:0\:x\le\:0\:\:\:\:\:\:\:\:\:\:\:\:\:\:\:\:\:\:\:\:\:\:\:\:\:\:\:\:\:\:\:\:\:\:\:\:\:\:\:\:\:\:\:\:\:\:\:\:\:\:\:\:\:\:$$

2 $$\:{F}_{{k}_{1}}^{t}=ReLU({b}_{{k}_{1}}+{\sum\:}_{i=1}^{n}({x}_{i}^{t}*{W}_{{k}_{1}\cdot\:i}\left)\right)\:\:\:\:\:\:\:\:\:\:\:\:\:\:\:\:\:\:\:\:\:\:\:\:\:\:\:\:\:\:\:\:\:\:\:\:\:\:\:\:\:\:\:$$

$\:{F}_{{k}_{1}}^{t}$ signifies the output outcome of 1st convolution layer, ReLU denotes an activation function, $\:{b}_{{k}_{1}}$ signifies the convolution kernel offset term of the first convolution layer, and $\:{W}_{{k}_{1}\cdot\:}$means item weight.

Maximum pooling has been chosen by the initial pooling layer that could decrease the network difficulty and preserve the essential features afterwards convolution. The formulation for the first pooling output outcome can be represented below:

3 $$\:{F}_{{m}_{1}}^{t}=max\left({F}_{{k}_{1}}^{t}\left({d}_{1}\right)\right)\:\:\:\:\:\:\:\:\:\:\:\:\:\:\:\:\:\:\:\:\:\:\:\:\:\:\:\:\:\:\:\:\:\:\:\:\:\:\:\:\:\:\:\:\:\:\:\:\:\:\:\:\:\:\:\:\:\:\:$$

whereas $\:{F}_{{m}_{1}}^{t}$ signifies the output outcome of initial pooling layer; $\:{F}_{{k}_{1}}^{t}\left({d}_{1}\right)$ represents $\:{d}_{1}^{\tau\:h}$ vector in initial convolution layer output, $\:{d}_{1}\in\:{m}_{1}.$

After the 2nd convolution, the eigenvector is attained from the initial pooling layer output outcome that is displayed below:

4 $$\:{F}_{{k}_{2}}^{t}=ReLU\left({b}_{{k}_{2}}+{\sum\:}_{{d}_{1}=1}^{m1}{F}_{{d}_{1}}^{t}*{W}_{{k}_{2}\cdot\:{d}_{1}}\right)\:\:\:\:\:\:\:\:\:\:\:\:\:\:\:\:\:\:\:\:\:\:\:\:\:\:\:\:\:\:\:\:\:\:\:\:\:\:$$

whereas $\:{b}_{{k}_{2}}$ represents the convolution kernel offset term in the 2nd convolution layer; $\:{W}_{{k}_{2}\cdot\:{d}_{1}}$ denotes the weight item.

The 2nd pooling layer output outcome can be represented below:

5 $$\:{F}_{{m}_{2}}^{t}=max\left({F}_{{k}_{2}}^{t}\left({d}_{2}\right)\right)\:\:\:\:\:\:\:\:\:\:\:\:\:\:\:\:\:\:\:\:\:\:\:\:\:\:\:\:\:\:\:\:\:\:\:\:\:\:\:\:\:\:\:\:\:\:\:\:\:\:\:\:\:\:\:\:\:$$

whereas $\:{F}_{{m}_{2}}^{t}$ signifies second pooling layer output of; $\:{F}_{{k}_{2}}^{t}\left({d}_{2}\right)$ represents $\:{d}_{2}^{th}$ vector output of 2nd convolution layer, $\:{d}_{1}\in\:{m}_{1}.$

At the end, the output eigenvector after double pooling and convolution is expressed below:

6 $$\:{F}^{t}=\left[{F}_{1}^{t},{F}_{2}^{t},.,{F}_{{m}_{2}}^{t}\right]\:\:\:\:\:\:\:\:\:\:\:\:\:\:\:\:\:\:\:\:\:\:\:\:\:\:\:\:\:\:\:\:\:\:\:\:\:\:\:\:\:\:\:\:\:\:\:\:\:\:\:\:\:\:\:\:\:\:\:\:\:$$

#### Multi-head attention (MA) mechanism

MA represents the attention mechanism variant, which can give weights to distinct locations or sections during sequential data processing. Also, it enables the method to concentrate on the various data on unidentified representation sub-spaces automatically. Additionally, the mechanism of MA depends on the Scaled Dot Product Attention computation section for attaining the weights of attention and the matrix of reconstructed attention that functions on keys (K), values (V), and queries (Q).it is represented below:

7 $$\:Attention\left(Q,K,\:V\right)=softmax\:\left(\frac{Q{K}^{T}}{\sqrt{{d}_{k}}}\right)y\:\:\:\:\:\:\:\:\:\:\:\:\:\:\:\:\:\:\:\:\:\:\:\:\:\:\:\:\:\:\:\:\:\:\:\:\:\:$$

whereas $\:{d}_{k}$ represents the dimensionality of the key; the softmax is utilized to achieve the attention weight matrix.

After that, the technique of MA achieves the attention representation on the head and combines the interchange outcomes served in the feedforward layer for additional calculation. The attention function can be implemented in corresponding with every proposed form$\:\:Q,\:K$, and V to produce the value of the output. The function of Concat integrates these values and, again, proposes to attain the concluding value. The formulation is represented below:

8 $$\:hea{d}_{j}=Attention\left(Q{W}_{i}^{Q},\:K{W}_{i}^{K},\:V{W}_{i}^{y}\right)\:\:\:\:\:\:\:\:\:\:\:\:\:\:\:\:\:\:\:\:\:\:\:\:\:\:\:\:\:\:\:\:\:\:\:\:\:\:\:\:\:\:\:$$

9 $$\:MultiHead\left(Q,K,\:V\right)=Concat\left(hea{d}_{1},\:hea{d}_{2},\:.,\:hea{d}_{h}\right){W}^{o}\:\:\:\:\:\:\:\:\:\:\:\:\:\:\:\:\:\:\:\:\:\:$$

$\:{W}_{i}^{y},{\:W}_{i}^{Q}$, and $\:{W}_{i}^{K}$ are weight matrices; $\:W$ represents the weight matrix used in the linear output function, and $\:hea{d}_{i}$ denotes head$\:\:i$ attention.

#### BiLSTM

The layer of BiLSTM integrates the bi-directional recurrent method in LSTM that enables the bi-directional handling of data sequences. Also, it could effectually study and seize the dependences in either backward or forward directions, while LSTM can be deceived to study dependences only in a forward direction. The BiLSTM layer can incorporate dual hid den layers in a united output. The forward $\:\left(\overrightarrow{{H}_{tp}}\right)$ and backward $\:\left(\overleftarrow{{H}_{tp}}\right)$ layer iterative computation of output sequences includes using the inputs of backward and forward correspondingly. Afterwards, the attained outputs (u) were combined before proceeding to the following layer. Next, the formulation can be presented as follows:

10 $$\:u_{{tp}} = \psi \:\left( {\vec{H}_{{tp}} ,\overset{\lower0.5em\hbox{$\smash{\scriptscriptstyle\leftarrow}$}}{H} _{{tp}} } \right)$$

This research iteratively fine-tunes the MA-CNN‐BiLSTM hyperparameter by utilizing the Grid Search technique, which is an automatic hyperparameters optimizer technique which may expansively search for the optimum amalgamation of hyperparameter for ML methods in the solution space. In the layer of CNN, the values of filters and convolutional layers are fixed as 2 and 16, correspondingly. Also, the kernel size in the convolutional layer has been fixed to $\:3 \times$, the pooling operations size is $\:2\times 2$, and the pooling layer type is average pooling. In the layer of the MA method, the dropout rate and heads are fixed at 6 and 0.3. In a layer of BiLSTM, the hyperparameter in the backward and forward layers is fine-tuned.

### Segmentation: Unet3+

**Fig. 5:**
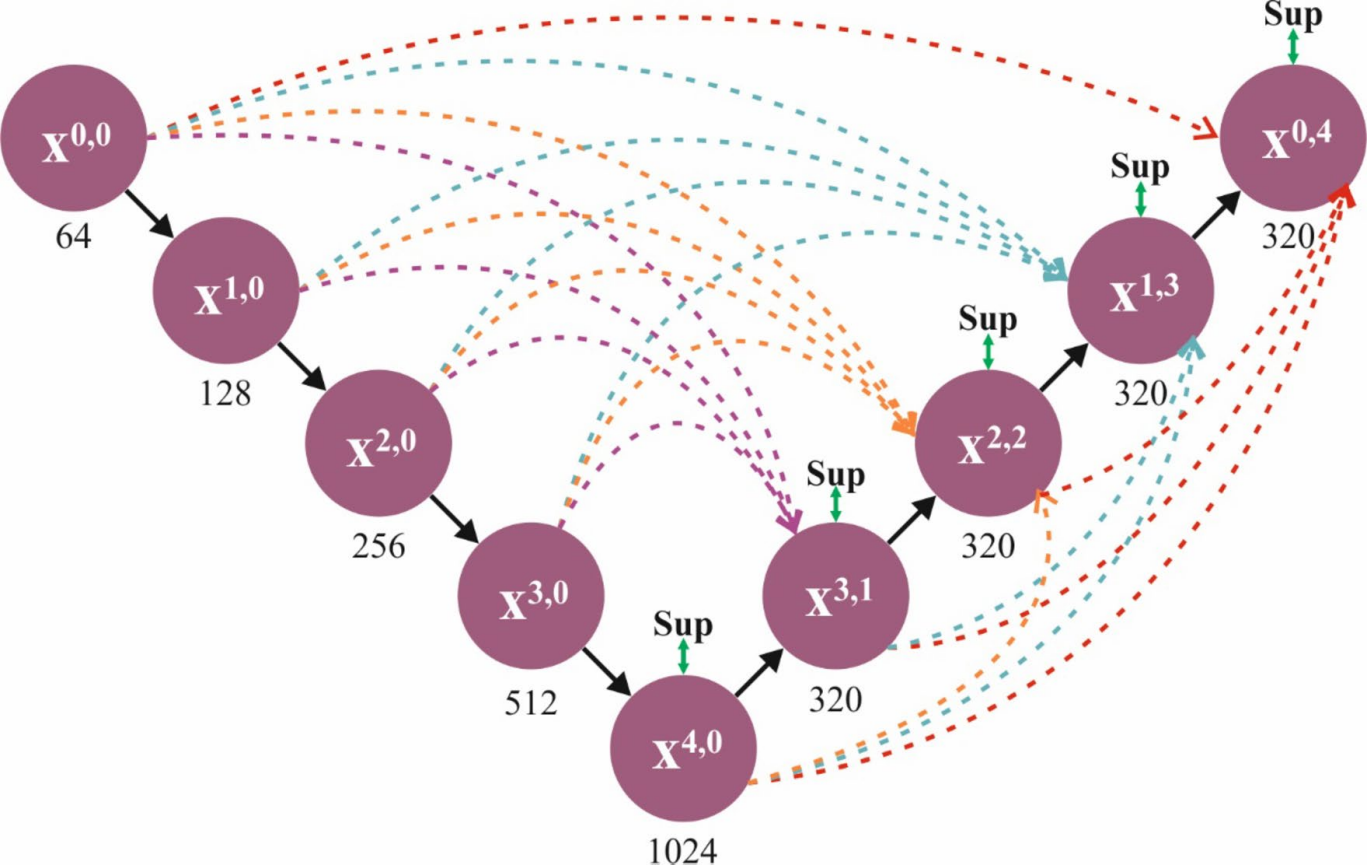
Structure of UNet3 + approach.

Moreover, the Unet3 + is employed to segment abnormal regions from the classified images^32^. This model was chosen due to its superior performance in handling complex image segmentation tasks, specifically in medical imaging, where precise delineation of abnormal regions is significant. Unlike conventional U-Net methods, Unet3 + presents deep supervision, which assists in improving the learning process and fine-tuning the segmentation accuracy at multiple scales. Its nested skip pathways allow for improved feature propagation and refinement, more precisely segmenting smaller or irregularly shaped lesions. Furthermore, Unet3 + is highly effective in dealing with imbalanced datasets, which is primarily a challenge in medical image segmentation. This ability of the model to retain high spatial resolution and its robustness in capturing both local and global features make it a robust choice over other segmentation methods. Also, its flexible architecture confirms efficient segmentation even in noisy or incomplete data, a common issue in clinical environments. UNet3 + contains five major sections: the full-scale skip connection module, the encoder module (down‐sampling), the full‐scale feature supervision module, the decoder module (up‐sampling), and the classification guidance module. Figure 5 illustrates the architecture of the UNet3 + model.

Encoder module: The encoded part is similar to UNet. Initially, the input image can be convolved twice through $$\:3\times\:3$$$$\:conV$$, succeeded by ReLU, BatchNorm2d. And then, the max-pooling function has been made, in other words, $$\:2\times\:2$$$$\:conV$$ by stride = 2. Down-sampling can no longer be done later in the 5th convolution layer (max-pooling process). While the $$\:3\times\:3$$ convoluted function influences the feature network, down-sampling (Max-pooling for down‐sampling) impacts the resolution. BatchNorm2d executes ReLU and data normalization. Max-pooling utilizes a $$\:2\times\:2$$$$\:conV$$ layer for feature extraction to minimize the feature mapping resolution through a factorization of 1.

Decoder module: This encoding part applies convolution and pooling to decrease the image size, which lessens the image resolution and produces some lost detailed information. During the decoder, the data of images can be consummated to a certain amount by double up-sampling, which rebuilds the image to its new dimension to classify every point of a pixel. Every decoding layer blends smaller‐scale feature mapping from the encoding, same‐scale feature mapping, and larger‐scale features from the decoding, and the featured mapping takes fine‐ and coarse‐grained semantics at complete measure. The smaller‐scale feature mapping from the encoding part reduces the dimension of feature mapping in supreme pooling and changes the channel counts from feature mapping in convolution. Same‐scale feature mapping from the encoding changes the channel counts from the feature mapping through convolution. Larger‐scaled features from the decoding part develop the dimension from the feature mapping by up‐sampling and transforming the dimension and channel counts from the feature mapping in convolution. Then, every feature mapping is joined through the channel stage to gain a feature mapping through unique channel counts. Then, BN$\:+$ReLU, convolution, and feature mapping from the decoder level are achieved to understand the complete‐scale featured fusion.

Full-scale jump connectivity module: It changes the connections between the encoding and decoding and the interior connections inside the decoding part. Every decoding level in UNet3 + includes same‐scale and feature mapping from the encoding and larger‐scale feature mapping from the decoding part, which is captured by the complete scale. Therefore, it reimburses the shortage of UNet and UNet + + for exploring adequate data from the full scale to gain a clean image from the position and targeted boundaries. Every decoding layer $\:{X}_{De}^{i}$ fuses feature mapping from various resources as formulation (1) demonstrations.

11 $$X_{{De}}^{i} = \left\{ {X_{{En}}^{i} ,~i = N~H\left( {\left[ {C\left( {D\left( {X_{{En}}^{K} } \right)} \right)_{{K = 1}}^{{i - 1}} ,\underbrace {{C\left( {X_{{En}}^{K} } \right)}}_{{_{{Scales:1^{{th}} \sim i^{{th}} }} }},\,\underbrace {{C\left( {u\left( {X_{{De}}^{K} } \right)} \right)_{{K = i + 1}}^{N} }}_{{Scales:\left( {i + 1} \right)^{{th}} \sim N^{{th}} }}} \right]} \right),~i = 1, \ldots ,N - 1~} \right\}~$$

$\:C(\cdot\:)$ offers a convolution process, and $\:H(\cdot\:)$ signifies a feature transmission method with a batch normalization, a convolution, and an activation function of ReLU. $\:D(\cdot\:)$ and $\:U(\cdot\:)$ denote down- and up‐sampling, and $\:\left[\cdot\:\right]$ characterizes concatenation operation.

Full-scale feature supervision module: Full‐scale, more profound supervision has been offered on UNet3 + to make an attack outputting supervision by the ground fact at every decoder level. This stage contains the subsequent operations: $$\:3\times\:3$$$$\:conV$$, sigmoid, and bi-linear up‐sampling. The particular operation of in-depth supervision: This final layer from the feature mapping produced by the feature accumulation method of every decoder level is nourished into the $$\:3$$x$$\:3$$ convolution layer, which a bi-linear up-sampling can then complement. The output is then gained by multiplying the segmented outcome attained next up‐sampling through the classifier module outcome 0 or 1. The multiplication outcome is exposed to handling sigmoid. The result achieved is intensely supervised output. The more profound supervision outcome is then inputted into the losing function.

Classification bootstrap module: To get more precise segmented outcomes, UNet3 + forecasts whether the inputting image comprises the targeted segmentation by adding a classification task. The deeper 2D tensor Encoding5 experiences a sequence of operations comprising Sigmoid, Convolution, Max-pooling, and Dropout. It ends up with dual values demonstrating the likelihood of consuming or not consuming a targeted segmentation. Using the finest semantic information, identification outcomes are additionally directed by the output of two stages for every cut side. With the assistance from the $\:Argmax$ function, the dual-dimension tensor has been changed into a singular output of $\:\{0,\:1\}$, with $\:0$ signifying absence and 1 demonstrating presence. Then, the individual classified outputs are increased by the side segmented outputs. Owing to the easiness of the dual task for classification, these modules gain precise classification outcomes by enhancing the binary cross‐entropy losing function.

### Parameter tuning: SCA model

**Fig. 6:**
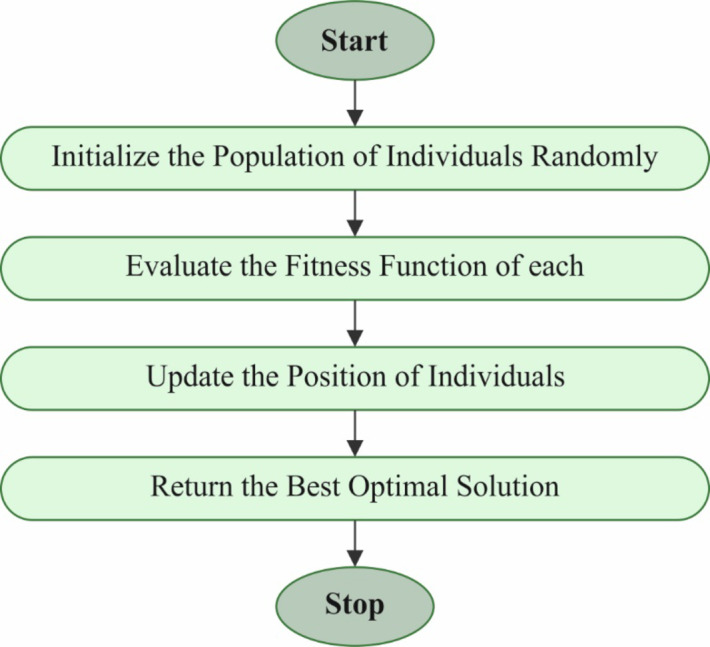
Workflow of the SCA model.

Finally, the SCA for the DL model’s hyperparameter tuning leads to enhanced performance^33^. This model is chosen for parameter tuning due to its robust capability to balance exploration and exploitation during optimization. Unlike conventional optimization techniques, SCA replicates the sine and cosine functions to search the solution space and avoid local minima effectually. This allows for more accurate and effectual tuning of model parameters, specifically in intrinsic DL models. The simplicity of the SCA, along with its minimal number of parameters to adjust, makes it computationally efficient, which is crucial for hyperparameter optimization in large-scale models. Moreover, it has exhibited superior convergence properties and robustness in optimizing non-linear and high-dimensional problems, making it an ideal choice over other optimization algorithms, such as genetic algorithm or particle swarm optimization. Figure 6 demonstrates the working flow of the SCA model.

The SCA has developed into a flexible optimizer model during the growth of optimization realm approaches. These mathematical features of cosine and sine functions have stimulated the making of SCA. It works with candidate solutions simultaneously as a population-based optimizer approach. These populations grow throughout iterations to improve solution qualities. To balance exploitation and exploration, SCA successfully directs problematic spaces in search of solutions. The exploration includes uncovering solution parts, whereas exploitation concentrates on refining current solutions. One of the basic features of SCA is its process of upgrading solutions by combining cosine and sine functions. By incorporating this function, complexity and randomness are presented in the optimizer process, allowing SCA to avoid becoming stuck at points instead of venturing into several parts of the solution spaces. Succeeding this is a sequence of expressions prescriptions for how locations are upgraded in the SCA method. Regarding either exploitation or exploration phases, it is vital to refer to Eqs. (12) and (13).

12 $$\:{X}_{i}^{t+1}={X}_{i}^{t}+{r}_{1}*sin\left({r}_{2}\right)*\left|{r}_{3}{P}_{i}^{t}-{X}_{i}^{t}\right|\:\:\:\:\:\:\:\:\:\:\:\:\:\:\:\:\:\:\:\:\:\:\:\:\:\:\:\:\:\:\:\:\:\:\:\:\:\:\:\:\:\:\:$$

13 $$\:{X}_{i}^{t+1}={X}_{i}^{t}+{r}_{1}*cos\left({r}_{2}\right)*\left|{r}_{3}{P}_{i}^{t}-{X}_{ii}^{t}\right|\:\:\:\:\:\:\:\:\:\:\:\:\:\:\:\:\:\:\:\:\:\:\:\:\:\:\:\:\:\:\:\:\:\:\:\:\:\:\:\:\:\:\:$$

In this setting, $\:{X}_{i}^{t}$ indicates the locations of the recent solution in the $\:{i}_{th}$ dimension throughout the iteration, with $\:{r}_{1}$, $\:{r}_{2}$, $\:{and\:r}_{3}$ indicating 3 randomly generated values. The placed point directs the location in the $\:{i}_{th}$ dimension, and $\:ii$ symbolizes absolute values. The implementations of this dual equation are interconnected as follows:

14 $$\:{X}_{i}^{t+1}=\{{X}_{i}^{t}+{r}_{1}*sin\left({r}_{2}\right)*\left|{r}_{3}{P}_{i}^{t}-{X}_{i}^{t}\right|,{r}_{4}<0.5\:{X}_{i}^{t}+{r}_{1}*cos\left({r}_{2}\right)*\left|{r}_{3}{P}_{i}^{t}-{X}_{ii}^{t}\right|,{r}_{4}\ge\:0.5\:\:\:\:\:\:\:\:\:\:\:\:\:\:\:\:\:$$

Now, $\:{r}_{4}$ denoted a randomly generated number from [0,1].

With an array of initial solutions generated randomly, this algorithm maintains the optimum solutions recognized throughout the method, allocating this for targeting points for following iterations. It then regulates another solution relative to these benchmarks. To guarantee a detailed investigation of the search space, these kinds of cosine and sine functions are upgraded in every process iteration. The optimizer procedure of the SCA is determined after it strikes the pre-established bounds of iterations. Still, another method to conclude the procedure can be applied, such as accomplishing several evaluations or reaching an accuracy level for the finest solution established.

The fitness range is a significant factor in manipulating the efficiency of SCA. The hyperparameter selection procedure includes the solution-encoded technique to assess the efficiency of the candidate solution. In this paper, the SCA reflects accuracy as the primary measure to project the fitness function (FF), expressed below in mathematical formulation.

15 $$\:Fitness\:=\:max\:\left(P\right)\:\:\:\:\:\:\:\:\:\:\:\:\:\:\:\:\:\:\:\:\:\:\:\:\:\:\:\:\:\:\:\:\:\:\:\:\:\:\:\:\:\:\:\:\:\:\:\:\:\:\:\:\:\:\:\:\:\:\:\:\:$$

16 $$\:P=\frac{TP}{TP+FP}\:\:\:\:\:\:\:\:\:\:\:\:\:\:\:\:\:\:\:\:\:\:\:\:\:\:\:\:\:\:\:\:\:\:\:\:\:\:\:\:\:\:\:\:\:\:\:\:\:\:\:\:\:\:\:\:\:\:\:\:\:\:\:\:\:\:\:$$

Here, TP and FP signify the true and false positive values.

## Result analysis and discussion

**Fig. 7:**
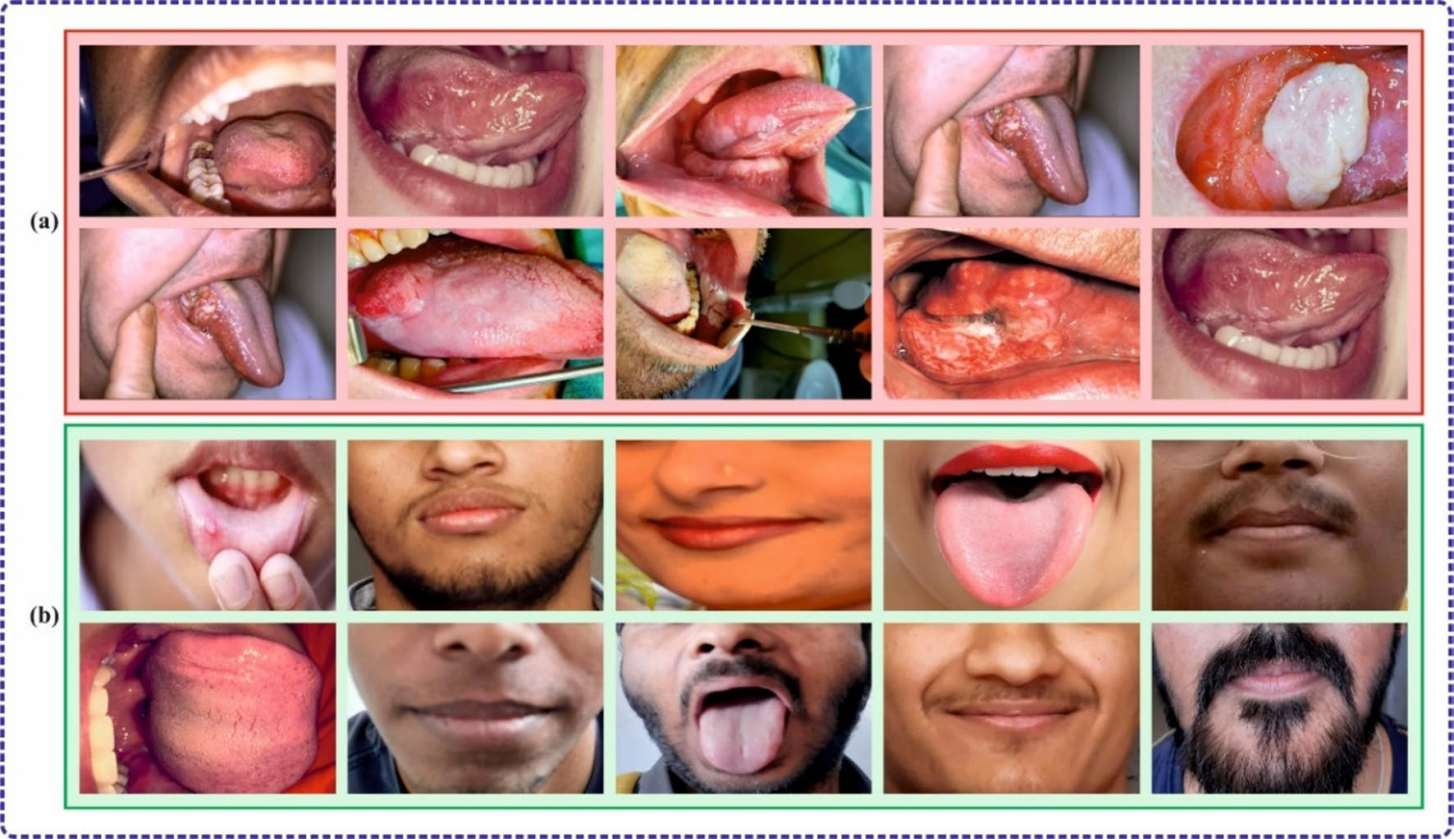
Sample images (**a**) cancerous (**b**) non-cancerous.

**Fig. 8:**
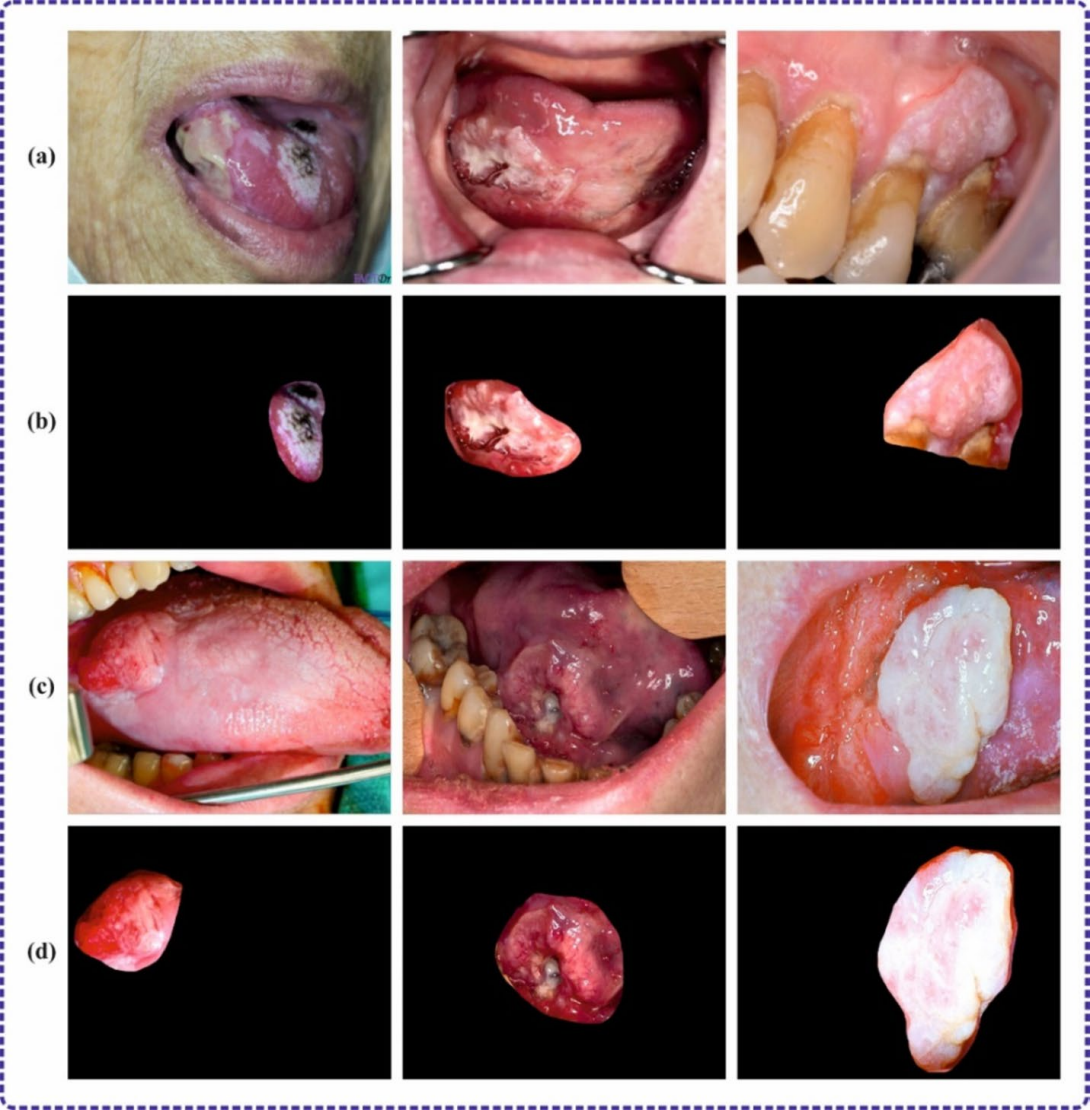
Sample images (**a** and **c**) cancerous (**b** and **d**) segmented.

In this section, the experimental validation of the DSLVI-OCLSC methodology is examined using the OC images dataset^34^. The dataset covers 131 instances under dual classes, as shown in Table 1. Figures 7 and 8 denote sample images.

**Table 1:** Details on dataset.

Classes	No. of instances
Cancer	87
Non-cancer	44
Total instances	131

**Fig. 9:**
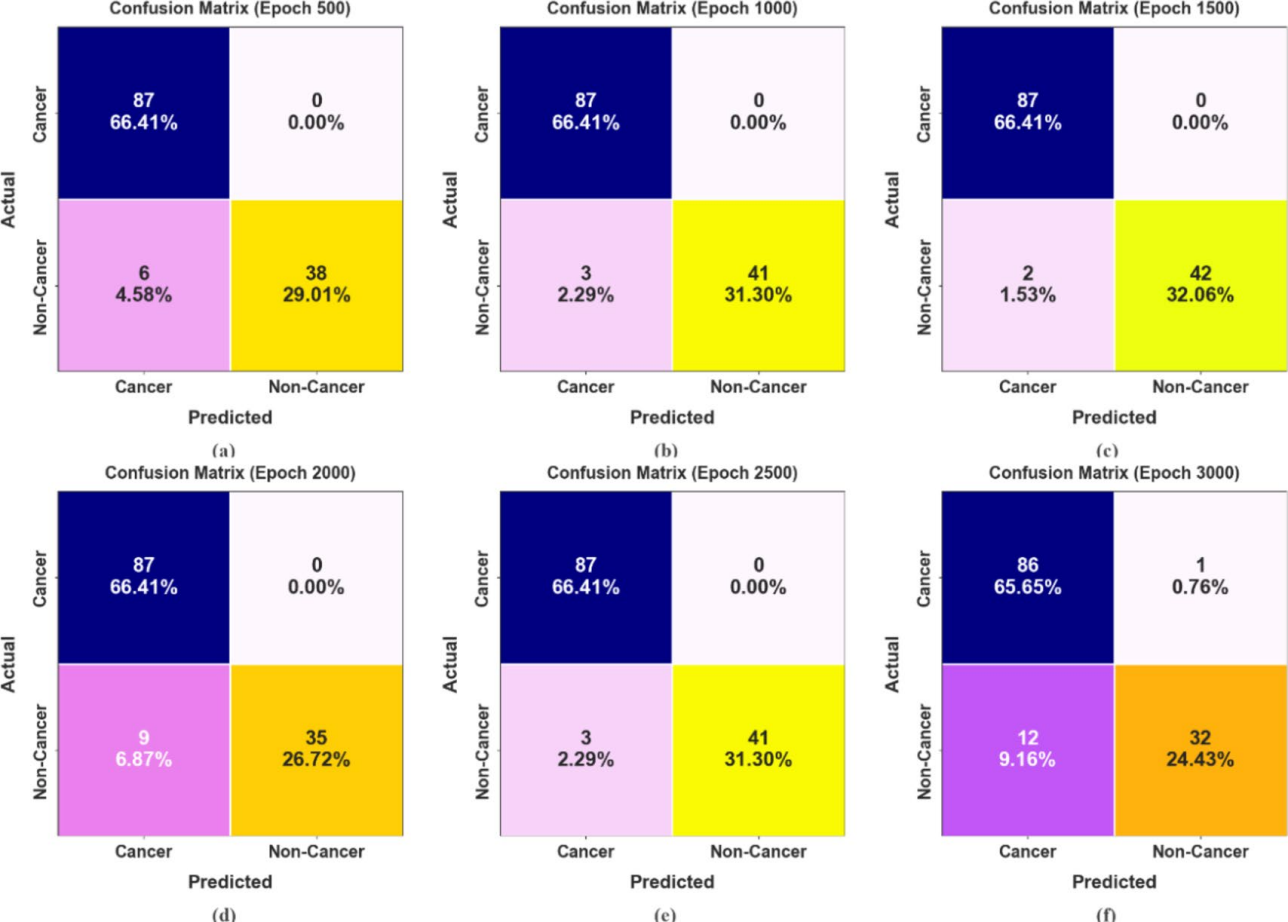
Confusion matrices of DSLVI-OCLSC model (**a**-**f**) Epochs 500–3000.

Figure 9 reports a set of confusion matrices formed by the DSLVI-OCLSC technique on dissimilar epochs. The results indicate that the DSLVI-OCLSC approach accurately recognizes and classifies dual classes.

**Fig. 10:**
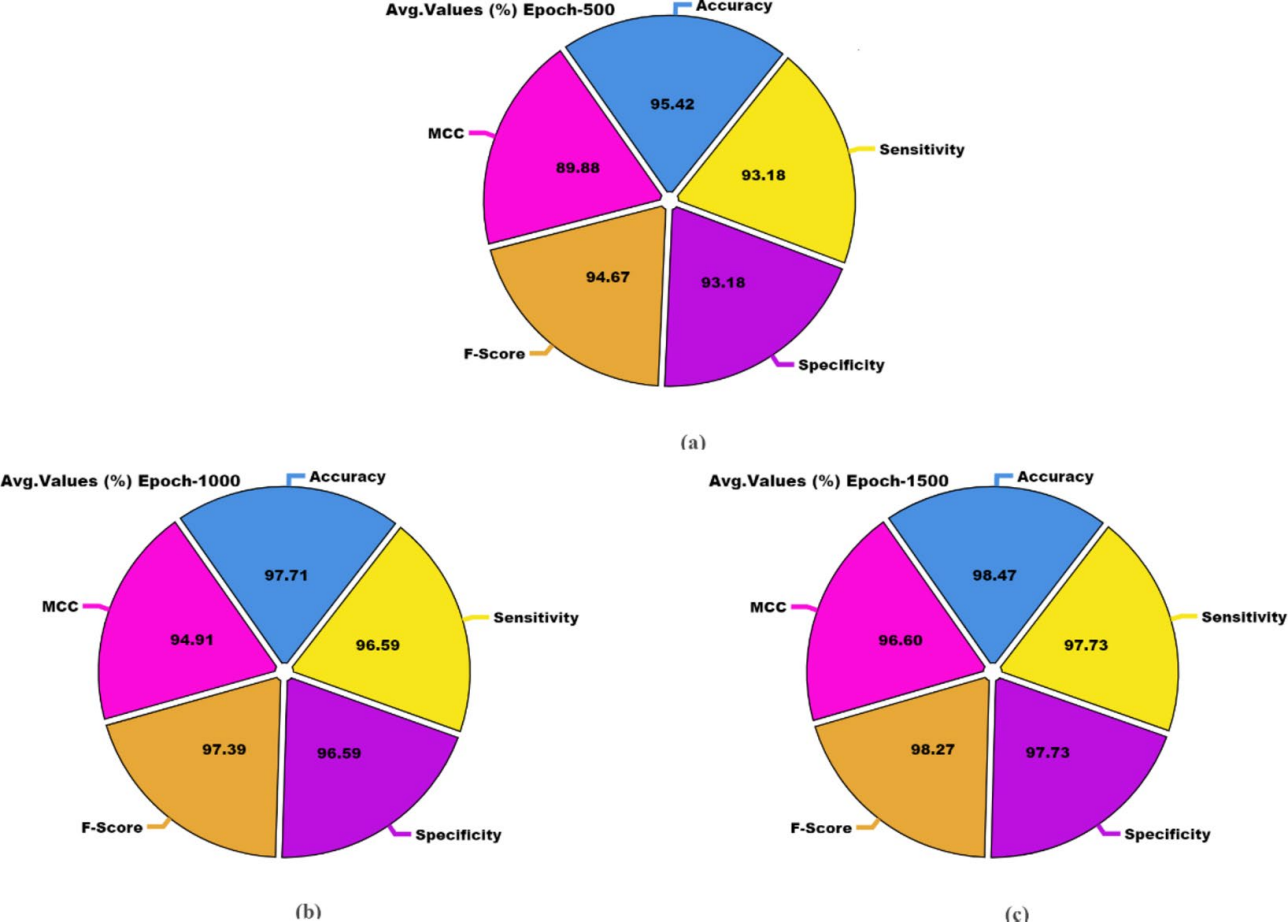
Average of DSLVI-OCLSC model under distinct epochs (**a**-**c**) epochs 500–1500.

The OC recognition outcomes of the DSLVI-OCLSC technique under dissimilar epochs are defined in Table 2. The table values state that the DSLVI-OCLSC technique correctly recognized all samples. Figure 10 shows the average outcome of the DSLVI-OCLSC approach under Epochs 500–1500. On 500 epochs, the DSLVI-OCLSC approach delivers an average $\:acc{u}_{y}$ of 95.42%, $\:sen{s}_{y}$ of 93.18%, $\:spe{c}_{y}$ of 93.18%, $\:{F}_{measure}$ of 94.67%, and MCC of 89.88%. Besides, on 1000 epochs, the DSLVI-OCLSC method gets an average $\:acc{u}_{y}$ of 97.71%, $\:sen{s}_{y}$ of 96.59%, $\:spe{c}_{y}$ of 96.59%, $\:{F}_{measure}$ of 97.39%, and MCC of 94.91%. Also, on 1500 epochs, the DSLVI-OCLSC method provides an average $\:acc{u}_{y}$ of 98.47%, $\:sen{s}_{y}$ of 97.73%, $\:spe{c}_{y}$ of 97.73%, $\:{F}_{measure}$ of 98.27%, and MCC of 96.60%.

**Table 2:** OC detection outcome of DSLVI-OCLSC model under distinct epochs.

Class	$\:Acc{u}_{y}$	$\:Sen{s}_{y}$	$\:Spe{c}_{y}$	$\:{F}_{measure}$	MCC
Epoch − 500					
Cancer	95.42	100.00	86.36	96.67	89.88
Non-Cancer	95.42	86.36	100.00	92.68	89.88
Average	95.42	93.18	93.18	94.67	89.88
Epoch − 1000					
Cancer	97.71	100.00	93.18	98.31	94.91
Non-Cancer	97.71	93.18	100.00	96.47	94.91
Average	97.71	96.59	96.59	97.39	94.91
Epoch − 1500					
Cancer	98.47	100.00	95.45	98.86	96.60
Non-Cancer	98.47	95.45	100.00	97.67	96.60
Average	98.47	97.73	97.73	98.27	96.60
Epoch − 2000					
Cancer	93.13	100.00	79.55	95.08	84.90
Non-Cancer	93.13	79.55	100.00	88.61	84.90
Average	93.13	89.77	89.77	91.84	84.90
Epoch − 2500					
Cancer	97.71	100.00	93.18	98.31	94.91
Non-Cancer	97.71	93.18	100.00	96.47	94.91
Average	97.71	96.59	96.59	97.39	94.91
Epoch − 3000					
Cancer	90.08	98.85	72.73	92.97	77.87
Non-Cancer	90.08	72.73	98.85	83.12	77.87
Average	90.08	85.79	85.79	88.04	77.87

**Fig. 11:**
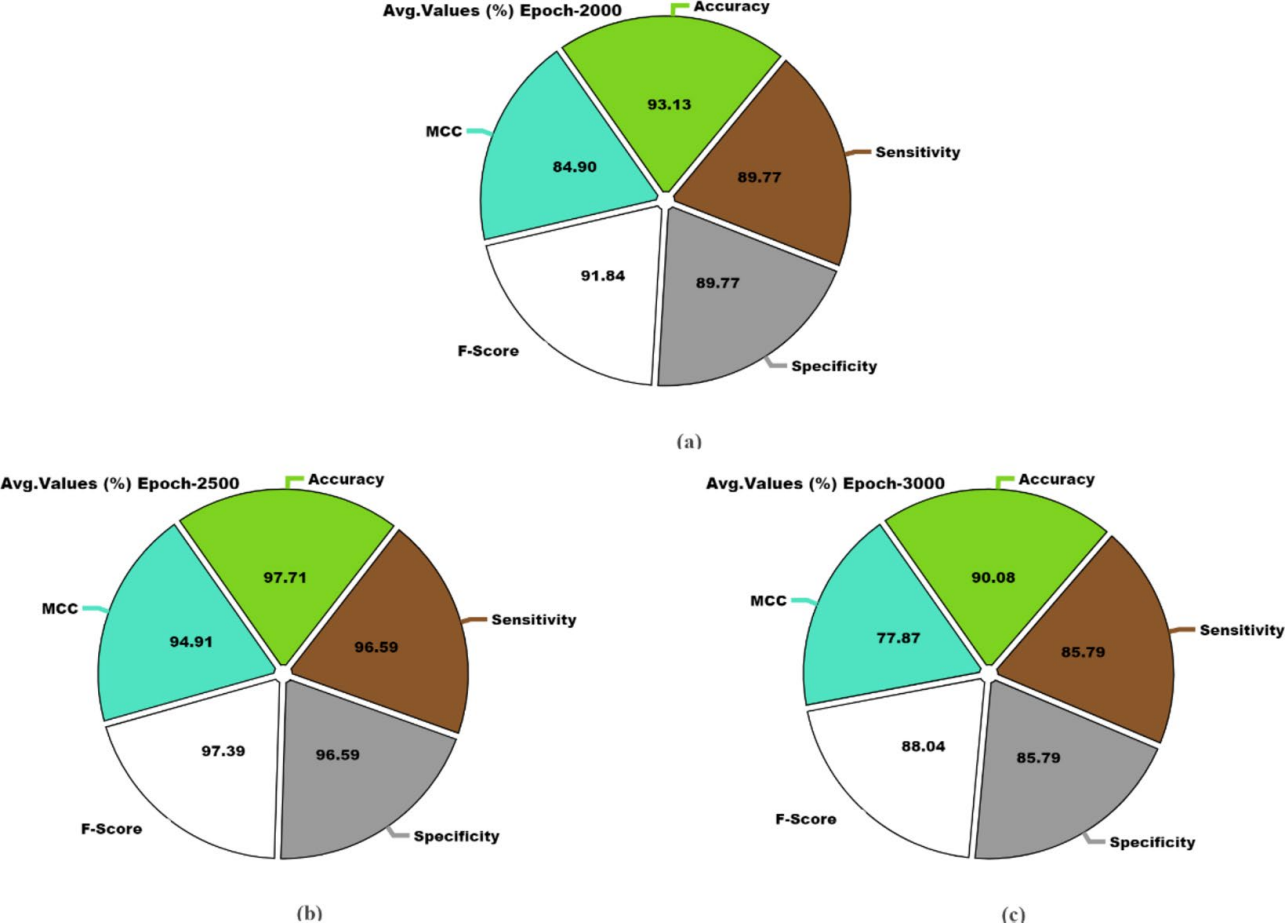
Average of DSLVI-OCLSC model under distinct epochs (**a**-**c**) epochs 2000–3000.

Figure 11 displays the average result of the DSLVI-OCLSC methodology under Epochs 2000–3000. Meanwhile, on 2000 epochs, the DSLVI-OCLSC methodology offers an average $\:acc{u}_{y}$ of 93.13%, $\:sen{s}_{y}$ of 89.77%, $\:spe{c}_{y}$ of 89.77%, $\:{F}_{measure}$ of 91.84%, and MCC of 84.90%. Besides, on 2500 epochs, the DSLVI-OCLSC technique delivers an average $\:acc{u}_{y}$ of 97.71%, $\:sen{s}_{y}$ of 96.59%, $\:spe{c}_{y}$ of 96.59%, $\:{F}_{measure}$ of 97.39%, and MCC of 94.91%. Besides, on 3000 epochs, the DSLVI-OCLSC technique presents an average $\:acc{u}_{y}$ of 90.08%, $\:sen{s}_{y}$ of 85.79%, $\:spe{c}_{y}$ of 85.79%, $\:{F}_{measure}$ of 88.04%, and MCC of 77.87%.

**Fig. 12:**
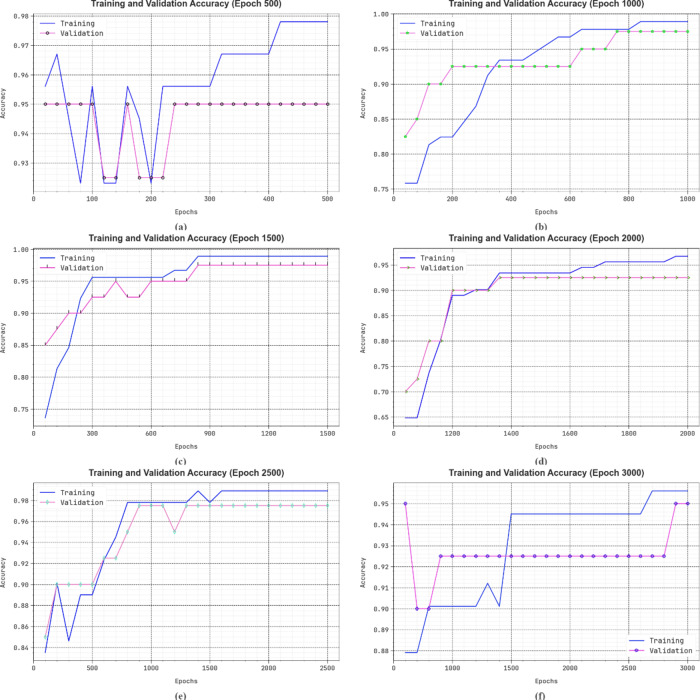
$\:Acc{u}_{y}$ curve of DSLVI-OCLSC technique (**a**-**f**) epochs 500–3000.

In Fig. 12, the training (TRA) and validation (VLA) accuracy results of the DSLVI-OCLSC model under diverse epochs are established. The accuracy values are calculated over a range of 0-3000 epochs. The figure emphasized that the TRA and VLA accuracy values display an increasing tendency, which alerted the capability of the DSLVI-OCLSC technique with enhanced performance over frequent iterations. Furthermore, the TRA and VLA accuracy rests closer over the epochs, which designates low least overfitting and shows the higher performance of the DSLVI-OCLSC technique, guaranteeing constant forecast on unseen samples.

**Fig. 13:**
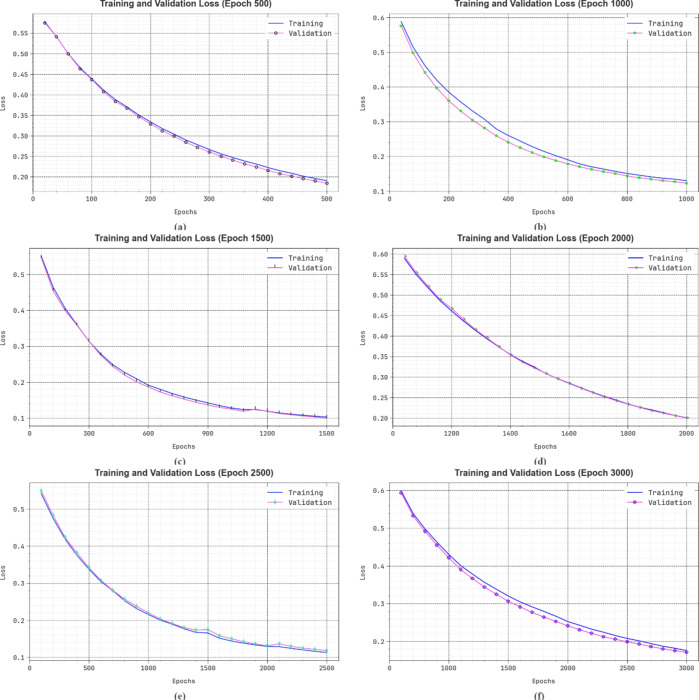
Loss curve of DSLVI-OCLSC technique (**a**-**f**) epochs 500–3000.

Figure 13 shows the TRA and VLA loss graph of the DSLVI-OCLSC methodology under dissimilar epochs. The loss values are figured throughout 0-3000 epochs. It is epitomized that the TRA and VLA accuracy values illustrate a declining tendency, notifying the ability of the DSLVI-OCLSC model to harmonize a trade-off between data fitting and generalization. The continual reduction in loss values further assures the heightened performance of the DSLVI-OCLSC approach and tunes the forecast outcomes over time.

**Fig. 14:**
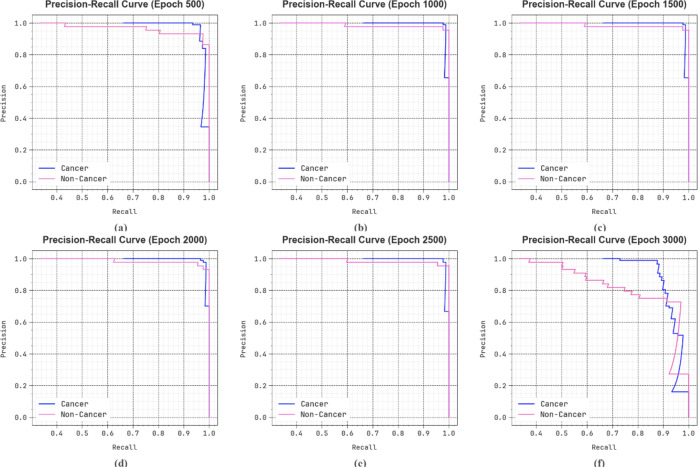
PR curve of DSLVI-OCLSC model (**a**-**f**) epochs 500–3000.

In Fig. 14, the precision-recall (PR) curve analysis of the DSLVI-OCLSC approach under diverse epochs offers an interpretation of its performance by plotting Precision against Recall for all the classes. The figure shows that the DSLVI-OCLSC approach constantly accomplishes enhanced PR values across dissimilar class labels, demonstrating its capability to preserve a significant portion of true positive predictions among every positive prediction (precision) while capturing a large ratio of actual positives (recall). The stable rise in PR results in every class portrays the efficiency of the DSLVI-OCLSC technique in the classification procedure.

**Fig. 15:**
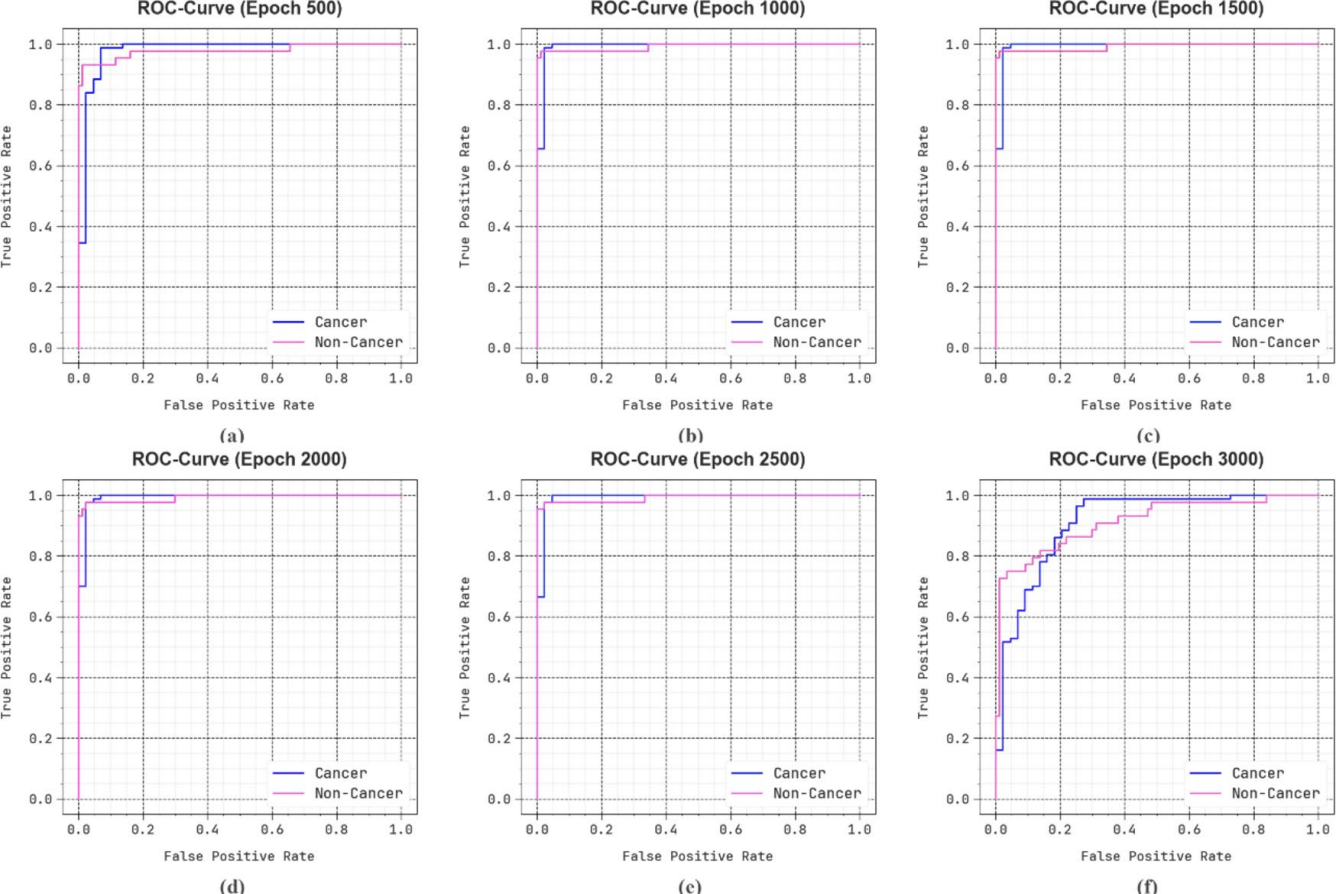
ROC curve of DSLVI-OCLSC technique (**a**-**f**) epochs 500–3000.

In Fig. 15, the ROC curve of the DSLVI-OCLSC method is studied. The outcomes indicate that the DSLVI-OCLSC technique reaches heightened ROC outcomes over each class under diverse epochs, signifying a significant ability to discern the classes. This dependable trend of improved ROC values over many classes designates the proficient performance of the DSLVI-OCLSC technique in forecasting classes, emphasizing the robust nature of the classification procedure.

The comparative analysis of the DSLVI-OCLSC technique with recent methodologies is confirmed in Table 3^35,36^. The simulation outcome identified that the DSLVI-OCLSC approach outperformed better performances.

**Fig. 16:**
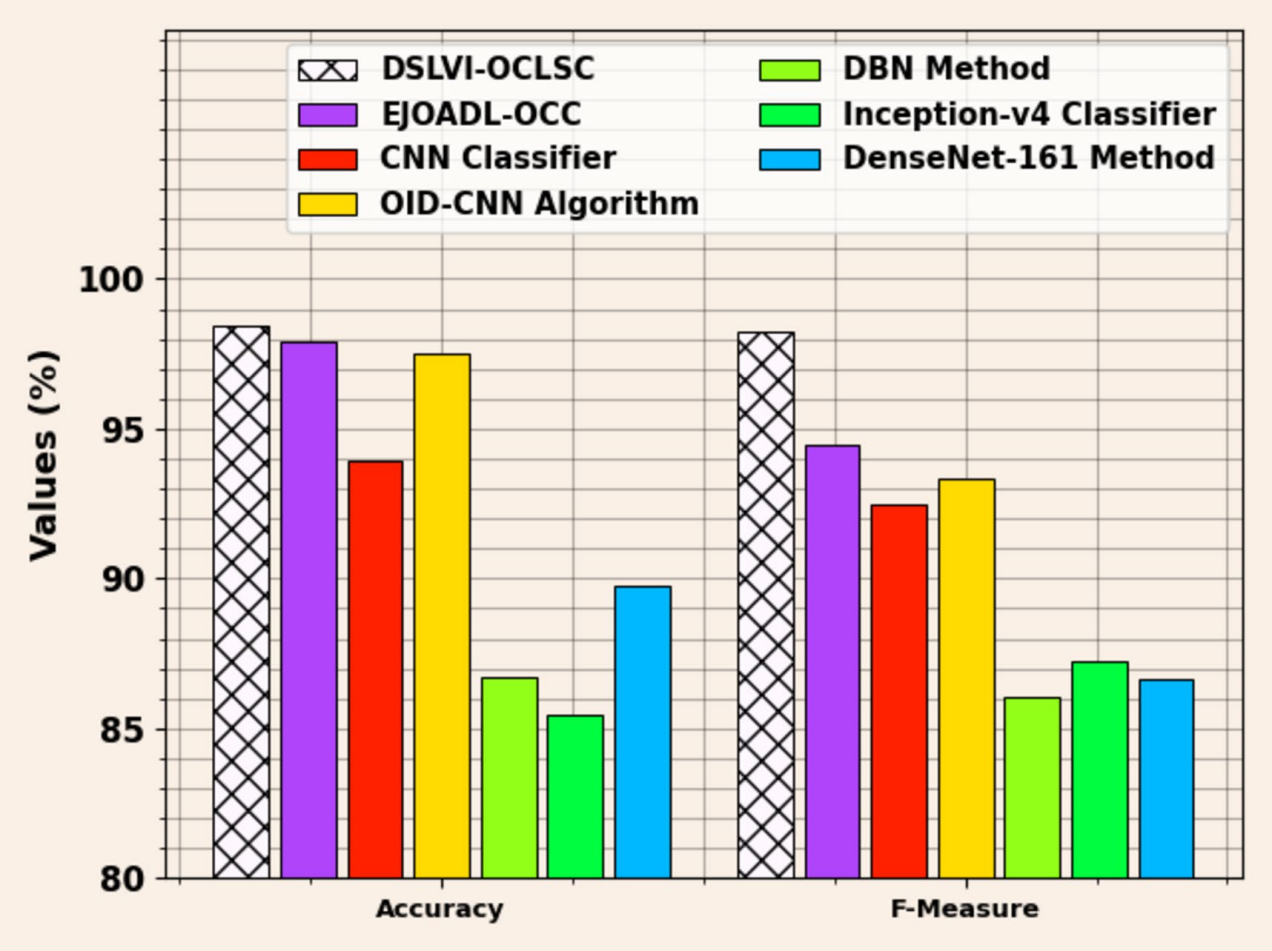
$\:Acc{u}_{y}\:$and $\:{F}_{measure}$ analysis of DSLVI-OCLSC approach with recent models.

Figure 16 compares the DSLVI-OCLSC technique with existing techniques regarding $\:acc{u}_{y}\:$and $\:{F}_{measure}.\:$Based on $\:acc{u}_{y}$, the DSLVI-OCLSC technique has advanced $\:acc{u}_{y}$ of 98.47%, whereas the EJOADL-OCC, CNN, OID-CNN, DBN, Inceptionv4, and DenseNet161 techniques have reduced $\:acc{u}_{y}$ of 97.88%, 93.96%, 97.53%, 86.68%, 85.44%, and 89.79%, respectively. While based on $\:{F}_{measure}$, the DSLVI-OCLSC methodology has a higher $\:{F}_{measure}$of 98.27% while the EJOADL-OCC, CNN, OID-CNN, DBN, Inception-v4, and DenseNet-161 methods have lesser $\:{F}_{measure}$ of 94.45%, 92.51%, 93.35%, 86.05%, 87.26%, and 86.66%, correspondingly.

**Fig. 17:**
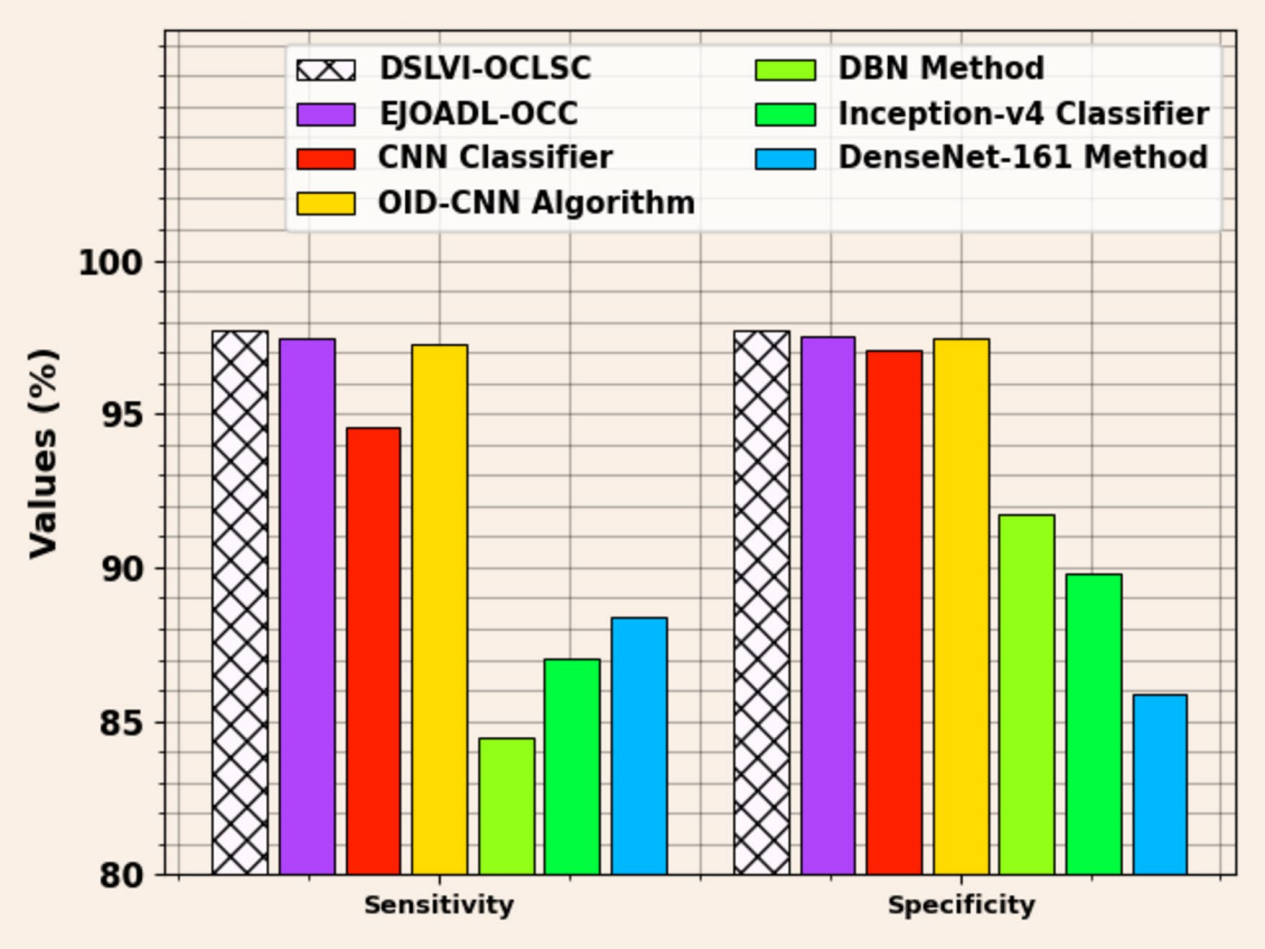
$\:Sen{s}_{y}\:$and $\:Spe{c}_{y}$ analysis of DSLVI-OCLSC approach with recent models.

Figure 17 displays a comparative analysis of the DSLVI-OCLSC approach with recent models for $\:sen{s}_{y}\:$and $\:spe{c}_{y}.\:$Based on $\:sen{s}_{y}$, the DSLVI-OCLSC approach has a higher $\:sen{s}_{y}$ of 97.73% while the EJOADL-OCC, CNN, OID-CNN, DBN, Inceptionv4, and DenseNet161 approaches have a lesser $\:sen{s}_{y}$ of 97.49%, 94.55%, 97.29%, 84.47%, 87.04%, and 88.36%, respectively. Whereas, based on $\:spe{c}_{y}$, the DSLVI-OCLSC approach has higher $\:spe{c}_{y}$ of 97.73% while the EJOADL-OCC, CNN, OID-CNN, DBN, Inceptionv4, and DenseNet161 approaches have lesser $\:spe{c}_{y}$ of 97.51%, 97.08%, 97.43%, 91.71%, 89.77%, and 85.85%, respectively.

**Table 3:** Comparative analysis of DSLVI-OCLSC approach with recent models^35,36^.

Methods	$\:Acc{u}_{y}$	$\:Sen{s}_{y}$	$\:Spe{c}_{y}$	$\:{F}_{measure}$
DSLVI-OCLSC	98.47	97.73	97.73	98.27
EJOADL-OCC	97.88	97.49	97.51	94.45
CNN classifier	93.96	94.55	97.08	92.51
OID-CNN	97.53	97.29	97.43	93.35
DBN	86.68	84.47	91.71	86.05
Inception-v4 classifier	85.44	87.04	89.77	87.26
DenseNet-161	89.79	88.36	85.85	86.66

**Fig. 18:**
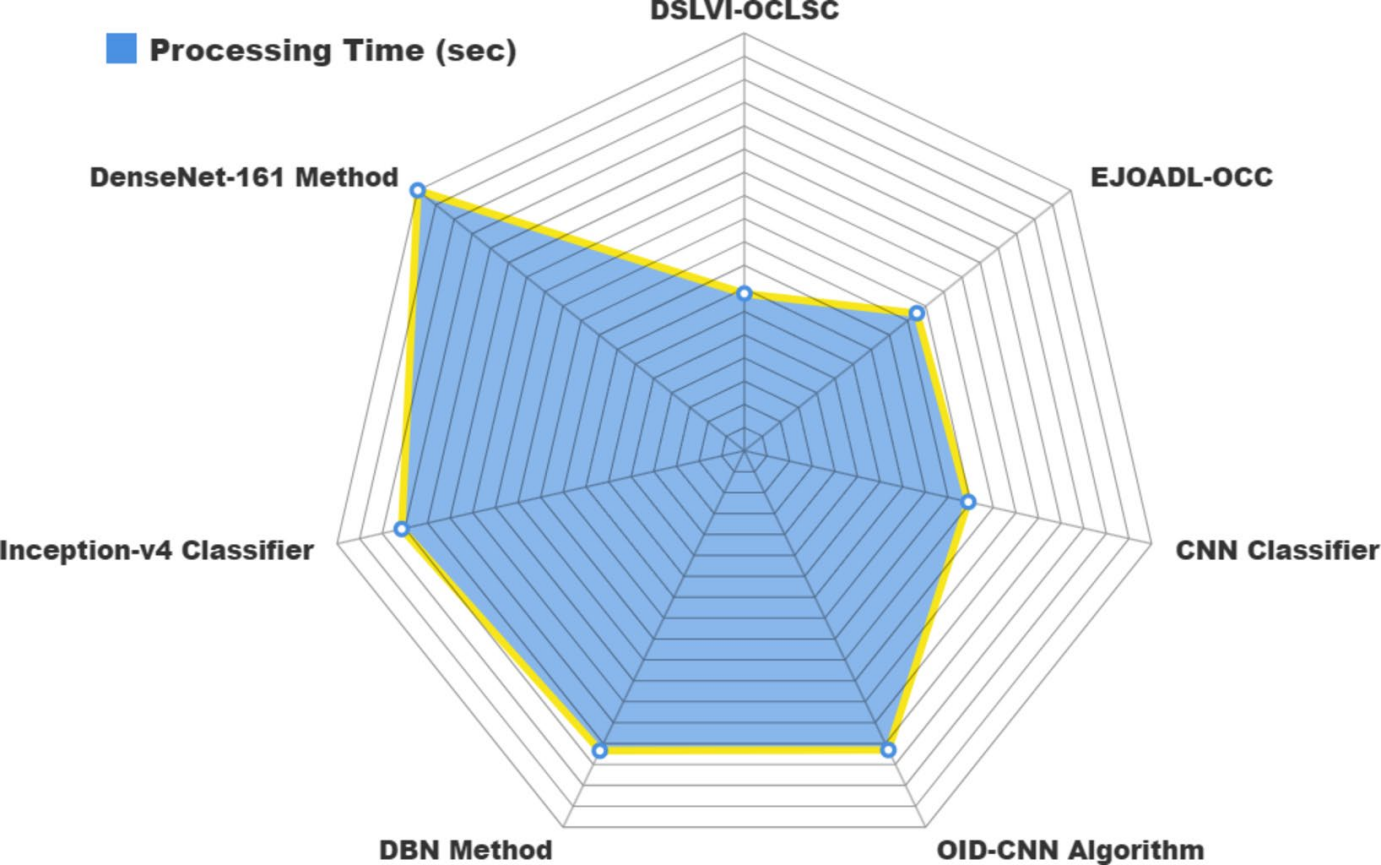
PT outcome of DSLVI-OCLSC technique with recent approaches.

In Table 4; Fig. 18, the comparative results of the DSLVI-OCLSC model are specified in terms of processing time (PT). The results suggest that the DSLVI-OCLSC method gets better performance. Based on PT, the DSLVI-OCLSC method delivers a lesser CT of 3.32s, whereas the EJOADL-OCC, CNN, OID-CNN, DBN, Inceptionv4, and DenseNet161 models get greater PT values of 4.66s, 4.85s, 7.01s, 7.03s, 7.42s, and 8.82s, respectively.

**Table 4:** PT outcome of DSLVI-OCLSC technique with recent approaches.

Methods	PT (sec)
DSLVI-OCLSC	3.32
EJOADL-OCC	4.66
CNN classifier	4.85
OID-CNN	7.01
DBN	7.03
Inception-v4 classifier	7.42
DenseNet-161	8.82

## Conclusion

In this paper, a DSLVI-OCLSC model is presented for medical imaging. The DSLVI-OCLSC model’s main objective is to enhance OC classification and recognition outcomes using medical imaging. To accomplish this, the DSLVI-OCLSC model utilized WF as a pre-processing technique to eliminate noise. In addition, the ShuffleNetV2 method is utilized for the group of higher-level deep features from input images. For OC recognition and identification, the MA-CNN‐BiLSTM approach was utilized. Moreover, the Unet3 + was employed to segment abnormal regions from the classified images. Finally, the SCA for the DL model’s hyperparameter tuning leads to enhanced performance. A wide range of simulations is implemented to ensure the enhanced performance of the DSLVI-OCLSC method under the OC images dataset. The experimental analysis of the DSLVI-OCLSC method portrayed a superior accuracy value of 98.47% over recent approaches. The limitations of the DSLVI-OCLSC method comprise the reliance on limited datasets, which may not capture the full variability of real-world cancer cases, potentially affecting the model’s generalizability. The study also encounters challenges associated with the high computational cost of training DL techniques, which can limit their scalability in clinical settings with large datasets. Furthermore, the model’s performance may degrade in the presence of low-quality images or artefacts, as current pre-processing methods may not handle all types of noise. Future work will incorporate additional imaging modalities, such as MRI or PET scans, to enhance the model’s ability to handle diverse medical imaging data. Improving noise reduction techniques, particularly for low-resolution and noisy images, will be a key direction in exploring advanced denoising methods. Optimization of computational efficiency for real-time deployment in clinical settings will also be prioritized. Integrating multi-modal data sources, including clinical history and genomic information, will further enhance diagnostic accuracy and provide a more comprehensive approach to disease detection.

## Data Availability

The datasets used and analyzed during the current study available from the corresponding author on reasonable request.
